# Recent progress of polydopamine nanoparticles as advanced antimicrobial nanomaterials

**DOI:** 10.3389/fbioe.2025.1678136

**Published:** 2025-10-17

**Authors:** Sandra Guzman-Sanchez, Niketan Patel, Alexandre Soares Rosado

**Affiliations:** Biological and Environmental Science and Engineering Division, King Abdullah University of Science and Technology, Makkah, Saudi Arabia

**Keywords:** polydopamine nanoparticles, antimicrobial resistance, smart nanomaterials, photothermal therapy, biofilm eradication

## Abstract

The global rise of antimicrobial resistance has driven the search for novel antimicrobial strategies with higher effectiveness than common antibiotics. Among various solutions, polydopamine nanoparticles (PDA NPs) have gained widespread attention owing to their biocompatibility, functional versatility, and responsiveness to environmental stimuli. This review summarizes recent advances in the synthesis, functionalization, and antimicrobial applications of PDA NPs, highlighting their potential as smart nanomaterials. PDA NPs exhibit intrinsic antimicrobial activity, large drug delivery capabilities, and excellent photothermal properties. Moreover, they can potentially eradicate biofilms; can be synergistically combined with other entities such as metal ions, antimicrobial peptides, and Fenton-like catalysts; and can provide *in vivo* models of bacterial infection. Despite these advantages, the widespread use of PDA NPs is limited by low synthesis reproducibility, insufficient accurate characterization, and lack of comprehensive biocompatibility assessment. Resolving these challenges is essential for fully comprehending and using the potential of PDA-based antimicrobial platforms. This review aims to explain the current landscape of PDA-based nanoformulations and to inspire future research toward clinically viable PDA-based nanoformulations.

## Graphical Abstract

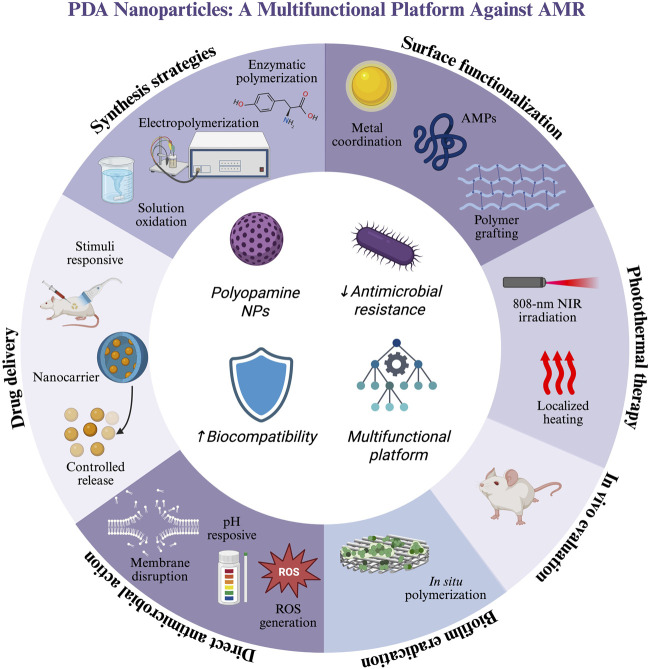

## 1 Introduction

Drug-resistant bacteria have emerged as a critical threat to global health. As drug-resistance development in bacteria has outpaced the development of new antibiotics, alternative antimicrobial approaches are urgent in demand. Antimicrobial resistance (AMR) is an evolutionary process through which microorganisms, such as bacteria, fungi, parasites, and viruses, resist antimicrobial treatments in humans and animals, thus increasing the difficulty of treating infections (Tang et al., 2023). The driving factors of AMR, dominated by the overuse and misuse of antibiotics, can be classified into four categories: spread of microorganisms owing to environmental problems such as rapid population growth and overcrowding; drug-related issues, such as fake drugs and easy access to over-the-counter medications; patient-related behaviors, such as self-medication; and healthcare-related factors, such as inappropriate prescriptions and overdosage (Salam et al., 2023).

The extent of this threat is reflected in the number of associated deaths and economic losses. In 2019 alone, approximately 1.27 million deaths worldwide were owing to drug-resistant bacterial infections. The highest mortality rates were recorded in sub-Saharan Africa and South Asia, where the impact of resistant infections is aggravated by limited access to healthcare and effective treatments (Murray et al., 2022). Without intervention, the global mortality rate is projected to reach to approximately 10 million deaths per year by 2050 (Chinemerem Nwobodo et al., 2022; Pulingam et al., 2022). Besides threatening public health, the increase in drug-resistant infections imposes substantial economic burdens, either directly (e.g., by increasing healthcare expenses) or indirectly (e.g., by reducing productivity and prolonging hospital stays). By 2050, AMR is projected to reduce annual productivity by 3% worldwide, corresponding to the economic losses of approximately $2.4–$6.9 trillion USD per year and an additional 8–24 million people living in poverty (Hall et al., 2018).

Nanomaterials have revolutionized the biomedical field, offering new possibilities for theranostics, drug delivery, regenerative medicine, and other applications. Physiochemical properties are magnified at the typical size of nanomaterials (0.1–100 nm; Singh et al., 2022; Yusuf et al., 2023). However, the lack of regulatory frameworks has prevented the widespread application of nanomaterials as therapeutic agents in biomedicine (Husen and Siddiqi, 2023). So-called “smart nanomaterials,” arising from the increasing demand for functionalized biomaterials that can mimic the behaviors of living organisms (Aflori, 2021), alter their own physical, chemical, and biological properties in response to environmental changes (Singh et al., 2022). Under external biological stimuli, smart nanomaterials self-modify their shape, surface area, size, and other properties (Aflori, 2021). Many smart nanomaterials are biomimetic and therefore usable in drug delivery systems and self-healing materials (Sharma and Hussain, 2020; Yoshida and Lahann, 2008). Such tools avail new opportunities in biomedical research and development, enabling enhanced drug therapies while reducing side effects. Personalized medicine is an especially exciting outcome of these opportunities (Michalska et al., 2018; Sharma and Hussain, 2020).

Smart nanomaterials include polydopamine nanoparticles (PDA NPs), introduced relatively recently in 2007. A PDA NP is an insoluble biopolymer resulting from the oxidative self-polymerization of dopamine, the PDA monomer (D’Ischia et al., 2014; Schanze et al., 2018). PDA possesses a complex chemical structure with multiple functional groups, such imines, amines, and catechol, that impart reactivity and adhesion properties. Consequently, PDA can adhere to numerous organic and inorganic materials (Tran et al., 2019). Unlike their inorganic counterparts, smart nanomaterials exhibit high biocompatibility, biodegradability, and photothermal activity; therefore, they are suitable for diverse biomedical applications (Battaglini et al., 2024; Gholami Derami et al., 2021).

**FIGURE 1:**
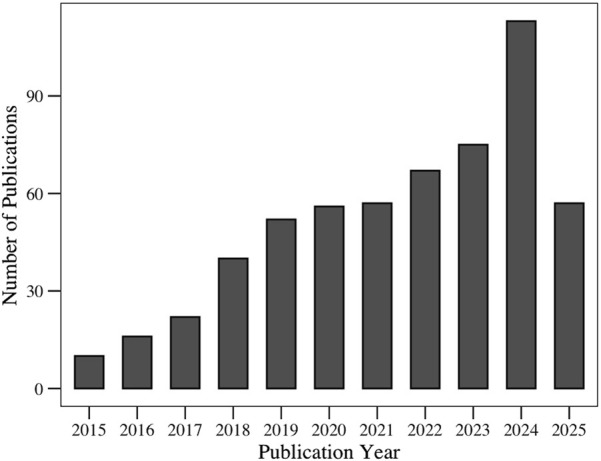
Evolution of number of publications related to the “antimicrobial applications of polydopamine,” retrieved from the Web of Science database.

In the past few years, the antimicrobial properties of polydopamine nanostructures have occupied an increasing share of the scientific literature. This growing trend reflects the increasing interest in PDA within the research community; in particular, PDA is regarded as a promising solution to the urgent global challenge of antimicrobial resistance. Although PDA has been extensively studied for its broad biomedical applications, most existing reviews have focused on general medical applications or hybrid systems that combine PDA with other materials. Few reviews have examined the inherent antimicrobial capabilities of PDA. As shown in Figure 1, the number of publications in this area is increasing each year, highlighting the relevance of such a review.

This review highlights the recent advancements in PDA-based antimicrobial strategies and identifies opportunities for future research, focusing on recent advancements in antimicrobial PDA NP agents and the potential of PDA NPs in combating AMR. This review discusses recent synthetic developments, the mechanisms underlying the antimicrobial activity of PDA NPs, and the different contexts of PDA NP applications. Moreover, it highlights the current limitations of nanomaterials, namely, the challenges of characterization, controlled delivery, and achieving high performance in biological systems. This review concludes with insights into future research directions in this area, aiming to guide the development of fully organic, biocompatible, and clinically viable PDA-based antimicrobial nanoplatforms. By presenting the latest progress, this review also aims to clarify the PDA NP role in designing next-generation strategies against AMR.

## 2 Synthesis of polydopamine nanoparticles

### 2.1 Solution oxidation method

**FIGURE 2:**
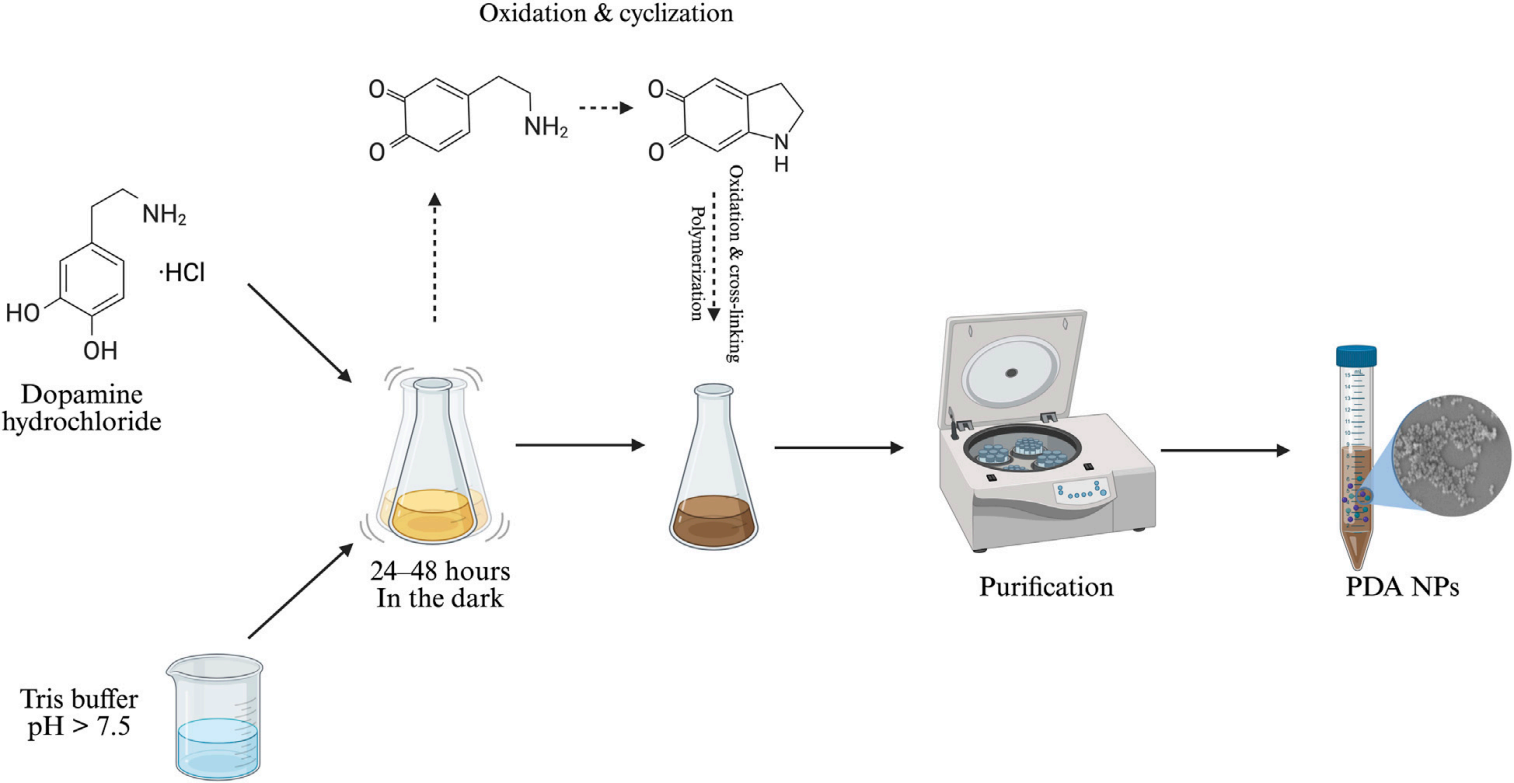
Synthesis of PDA NPs (image prepared using BioRender.com).

PDA NPs are usually synthesized through the oxidative self-polymerization of dopamine hydrochloride under mildly alkaline conditions, with dissolved oxygen acting as the oxidizing agent. This approach has been popularized for its simplicity and versatility (Ren et al., 2024). In general, dopamine hydrochloride is dissolved in an alkaline solution and the pH is adjusted to 7.5 or higher using a buffer (typically tris hydrochloride or sodium bicarbonate) or ammonia solution (Browne et al., 2022; Cicogna et al., 2023; Costa et al., 2024; Cui et al., 2024; 2025; Shumbula et al., 2022; Souza et al., 2025; Zhao B et al., 2024). The reaction proceeds under adjusted conditions at 25 °C or slightly higher (e.g., 37 °C) (Z. P. Li et al., 2022), with constant shaking over a fixed duration (24–48 h) to enable self-polymerization as shown in Figure 2 (Bano et al., 2023; Browne et al., 2022; Costa et al., 2024; Cui et al., 2024; 2025; S. Li et al., 2025; Qi et al., 2022; Shumbula et al., 2022; Souza et al., 2025; Wang H et al., 2024; Wang R et al., 2024; Wang H et al., 2024; Yu et al., 2022; Zhao B et al., 2024).

Polydopamine formation is initiated under mild alkaline conditions in the presence of dissolved oxygen to encourage the spontaneous oxidation of dopamine. The mechanism begins when dopamine oxidizes to the reactive compound dopamine–quinone. Facilitated by the availability of oxygen, this process is further accelerated at higher pH levels (Batul et al., 2023; Cai et al., 2024; Kensel Rajeev et al., 2024). A series of intramolecular transformations—intramolecular cyclization, oxidation, and crosslinking—lead to the insoluble and melanin-like PDA polymer. However, despite extensive studies, the exact polymerization mechanism remains incompletely understood, as researchers continue to debate the details of its polymerization pathway. The process is complicated by multiple competing reactions, shifts in protonation states influenced by pH, and the coexistence of several reactive species. This complexity not only makes the chemical structure of PDA heterogeneous, but also difficult to characterize with precision (Batul et al., 2023; Cai et al., 2024; Shumbula et al., 2022; Souza et al., 2025; Wang S et al., 2024; N. Xu et al., 2023; Yu et al., 2022).

Variations of this method use water-ethanol mixtures, followed by the addition of ammonium hydroxide to raise the pH. Ethanol affects the nucleation and growth of PDA NPs, allowing precise control over the NP formation (Cui et al., 2024; 2025; Wu et al., 2023). This process can be adapted to the co-formation or encapsulation of other materials and is suitable for biomedical, environmental, and materials science applications.

### 2.2 Electropolymerization of dopamine

The highly controlled electropolmerization method, creating PDA films on substrates or nanopores in the PDA matrix, offers numerous advantages over the solution oxidation method (Varol et al., 2023). The electropolymerization method oxidizes the dopamine monomer to electropolymerized polydopamine (ePDA) (Wang L et al., 2024), beginning with the removal of electrons from the catechol groups in dopamine when a potential difference is applied to the working electrode. The resulting current leads to the formation of dopamine–quinone. The activated benzoic ring then chemically interacts with the amine group through different reactions, ultimately forming an insoluble precipitate of PDA NPs on the surface of the working electrode (Marchesi D’Alvise et al., 2023).

Unlike traditional solution oxidation, electropolymerization can precisely control the thickness of nanometer-scale polymer coatings by adjusting the time and number of potential cycles (Varol et al., 2023). In addition, this technique produces uniform and high-quality films that seamlessly coat the substrate and deliver high performance (GhavamiNejad et al., 2015). The deposited films are adhesive, biocompatible, and can be functionalized with diverse molecules or efficiently immobilized nanoparticles to increase their antimicrobial properties (Almeida et al., 2021). The film thickness and composition are further influenced by pH, affecting the properties of PDA films during the electropolymerization process. Variations in pH can alter the deposition rate, structural arrangement, and overall characteristics of the resulting material (Marchesi D’Alvise et al., 2023). Alkaline conditions improve the efficiency of this type of synthesis, thus yielding thicker PDA layers. A change in pH directly influences the coating thickness by allowing precise control over the polymerization process of PDA, thereby enabling the tunability of properties such as layer thickness and uniformity. Furthermore, the presence of metal ions can augment the stability of the PDA film, thus strengthening the coating (Polaczek et al., 2024).

Its primary applications include creating antibacterial coatings, serving as an intermediate layer for molecule attachment, and regulating cell-surface interactions (Caniglia et al., 2023; Larrieu et al., 2024; Wang Y et al., 2024). However, this method has specific limitations as it requires conductive substrates, restricting its use compared to dopamine self-polymerization, which can coat nearly any surface. The method is primarily for surface-level modification, is less suitable for bulk material functionalization (Caniglia et al., 2023; 2024; Wang Y et al., 2024).

### 2.3 Enzymatic oxidation

Enzymatic oxidation catalyzes the polymerization using an oxidative enzyme such as laccase or tyrosinase (Magalhães et al., 2022; H. Zhang et al., 2020). Unlike the self-polymerization approach, requiring high alkalinity and long reaction times, the enzymatic method achieves fast polymerization under mild conditions, which broadens its range of substrates and applications (F. Li et al., 2018; Magalhães et al., 2022). For instance, one study found that laccase-catalyzed polymerization optimally proceeds at pH 5.5 °C and 30 °C with an initial dopamine concentration of 2 mg/mL; however, it remains more efficient than the conventional method over a broader pH range (4.5–7.5). At more alkaline pH, the performance is degraded by hydroxide interference at the active site of the enzyme (Magalhães et al., 2022).

In this approach, dopamine hydrochloride is dissolved in an aqueous buffer such as sodium acetate and the pH is adjusted to favor enzyme activity. After adding laccase, the solution is stirred at a controlled temperature. The polymerization is monitored visually (by observing changes, typically the darkening of the solution) and spectrophotometrically (by tracking the absorbance peaks around 305 and 480 nm denoting the intermediate species involved in PDA formation). Interestingly, the structure of enzymatically oxidized PDA differs from that of traditionally oxidized PDA. For instance, the dopamine units in enzymatically derived polymers are joined only by ether bonds. The resulting PDA films are generally more homogeneous, compact, and stable, with high reactivity and numerous amine-terminated molecules available for functionalization (F. Li et al., 2018; Magalhães et al., 2022). Moreover, dopamine polymerization has been catalyzed by tyrosinase, another oxidative enzyme that forms PDA in hydrogel matrices (for example, magnetic alginate–PDA beads), *in situ*. Here, PDA performs as a crosslinker and covalently bonds with amino groups to inhibit enzyme leakage (H. Zhang et al., 2020).

The enzymatic polymerization method offers several advantages over the conventional oxidation approach. Spontaneous oxidation in the traditional method is slow, requires alkaline conditions, and does not guarantee the stability of PDA. By contrast, enzymatic polymerization is fast, well-controlled, and proceeds under lower pH conditions, thus rendering it suitable for systems sensitive to high pH. Under optimized conditions, the enzymatic method achieves a much higher polymerization efficiency than the conventional process and is potentially applicable to various applications including surface coatings, biomedical materials, and enzyme immobilization.

## 3 PDA functionalization strategies

### 3.1 Metal-ion coordination and deposition

PDA exhibits a rich surface chemistry with catechol, amine, and imine functional groups that can be coordinated with metals. Therefore, PDA NPs can be functionalized with numerous metallic ions (Cu^2+^, Fe^3+^, Fe^2+^, Ti^4+^, Mn^2+^, Zn^2+^, and others) through coordination or deposition (Siciliano et al., 2022; Wang L et al., 2024; Z. Wang et al., 2020a).

The surface catechol groups on PDA NPs can stably coordinate with metal ions through chelation (Meng et al., 2018). Currently, metal ions are integrated into the PDA NPs matrix using the following three main methods:

1. Postdoping/postcoordination: Already synthesized PDA NPs are directly added to a solution containing a considerable excess of metal ions. This process requires a long exposure time for high functionalization (Wang L et al., 2024; Z. Wang et al., 2020b).
2. Predoping/precoordination: Dopamine monomers are copolymerized with metal ions in an alkaline environment. During polymerization, metal ions are continuously added to the growing PDA NPs structure, yielding NPs with a higher metal-ion content than those obtained by the postdoping process (Wang L et al., 2024; Z. Wang et al., 2020b).
3. Metal-ion exchange: PDA NPs are preloaded with specific metal ions using a preloading technique. The loaded ions can be exchanged with other desirable metal ions without causing colloidal instability or aggregation (Wang L et al., 2024; Z. Wang et al., 2020b).

Metal ions (most notably silver and gold) can also be incorporated into PDA NPs via metal deposition. This method exploits the redox capacity and strong binding affinity of PDA toward metal ions (e.g., Ag^+^, Au^3+^, and Cu^2+^). Subsequently, the ions are reduced to their elemental metallic state, thus generating noble metal NPs, metal oxide NPs, or metal sulfide NPs (H. Li et al., 2020; Z. Wang et al., 2020b).

Several studies have suggested that functionalization with metal ions can improve the catalytic activity, DNA adsorption, and controlled release of PDA NPs, promising applications ranging from cancer therapy to catalysis and biosensors. For instance, Wang S et al. (2024) combined the properties of metal ions with the biocompatibility of PDA, obtaining a metal-coordinated PDA structure with improved antimicrobial performance. They added silver nitrate to a preformed PDA solution, forming silver nanoparticles (Ag NPs) *in situ* through the predoping strategy. They observed that the catechol groups in PDA act as reducing agents, converting Ag^+^ ions into elemental silver, which binds to nitrogen and oxygen active sites and grows uniformly on the PDA surface. The resulting PDA–Ag-coated surfaces obviously reduced bacterial attachment and generated a strong antibiofilm effect. The antimicrobial performance was attributed to the direct antimicrobial effects of silver. When stabilized within PDA adhered to the antimicrobial surface, silver atoms can directly interact with and disrupt bacterial cells (Wang S et al., 2024).

### 3.2 Polymer grafting

The main strategies for grafting polymers onto PDA-coated surfaces are *grafting-to* and *grafting-from*. The first approach involves the separate synthesis of polymers in solution, which are later attached to the modified PDA surface. This method can be advantageous when the polymers must be thoroughly characterized before grafting because it can precisely control diverse properties such as molecular weight, polydispersity, and functionality. Nevertheless, steric hindrance limits the layer thickness to 10 nm or less because the preattached chains obstruct further polymer grafting (Teunissen et al., 2023).

The *grafting-from* method involves the direct *in situ* polymerization of monomers from initiators immobilized on the PDA surface (Nothling et al., 2022). This process typically begins with preparing a thin layer of PDA synthesized via the solution oxidation method. Polymerization initiators are covalently attached to PDA NPs. 2-Bromoisobutyryl bromide (BIBB) is commonly employed as the initiator because it reacts with the catechol and amine groups in PDA through nucleophilic acyl substitution, forming ester and amide bonds, respectively. BIBB-bound PDA provides surface-bound initiators for subsequent polymer growth (Hemmatpour et al., 2023). Subsequently, monomers are directly polymerized from the NP surface (Nothling et al., 2022). Afterward, the growth of polymer chains can be controlled using techniques such as atom transfer radical polymerization (Hemmatpour et al., 2023; Xiong and Ulbricht, 2024).

Polymer grafting onto PDA nanoparticles is a versatile approach for modifying PDA properties, especially in antimicrobial applications. For instance, Larrieu et al. (2024) coated polyurethane foams with PDA and further grafted them with silver NPs, obtaining a filter with effective antimicrobial activity against bacteria such as *Staphylococcus aureus* and *Escherichia coli* (Larrieu et al., 2024). PDA-based polymer grafting has also been explored in studies of drug delivery systems; for example, PDA-functionalized nanocarriers grafted with stimuli-responsive polymers exhibit controlled drug-release properties and are promising candidates for targeted therapies (Hemmatpour et al., 2023). PDA NPs can be modified with various functional polymers, highlighting their potential as advanced materials in biomedical and environmental applications.

### 3.3 PDA as a surface coating material

The strong adhesiveness of PDA is widely exploited in surface coating materials. PDA coatings are typically formed via the oxidative self-polymerization method; however, other methods are also employed. In the common dip-coating method, a substrate is immersed in a solution containing dopamine under alkaline conditions, inducing the self-polymerization of dopamine and the formation of a uniform dark-colored film that strongly adheres to organic and inorganic surfaces (Saikawa et al., 2024). After dipping, the coated material is typically washed to remove any unreacted dopamine. In some cases, the material is heat-treated to boost its durability (Behzadinasab et al., 2023). Electrochemical methods such as cyclic voltammetry or pulsed deposition can tune the properties of PDA, forming the desired material to be deposited on a preformed PDA layer (Caniglia et al., 2023; 2024). Various materials can be coated with PDA, including metals, e.g., titanium (Peng et al., 2025), magnesium alloy (Liu et al., 2025), gold (Caniglia et al., 2023), semiconductors, ceramics, polymers (polyvinylidene fluoride (He et al., 2023), polyurethane (Kan et al., 2025), polypropylene (e.g., Cicogna et al., 2023), and hydroxyapatite (Erdem et al., 2022).

For example, X. Zhao et al. (2023) fabricated nanopillar arrays as a mechanobactericidal nanostructured surface on polypropylene. They immersed the substrate in a dopamine HCl solution at pH 8.5, typically for 24 h in the dark, forming a uniform PDA layer. Air-plasma pretreatment increased the surface reactivity and hydrophilicity of the surface, thus improving the uniformity of the PDA layer. The obtained film was less than ∼10-nm thick and preserved the physical morphology and hence the mechanical bactericidal properties of the nanostructure. However, the introduction of hydroxyl and amino groups altered the surface chemistry, improving the photothermal antibacterial activity of the material.

As another example, Lu et al. (2015) coated PDA NPs on modified silk fibers, which are naturally prone to bacterial contamination. When silk is immersed in dopamine solution, the PDA coating formed on its surface provides a matrix for the *in situ* synthesis of AgNPs. Owing to its redox and metal-chelating capabilities, PDA readily adsorbs silver ions and directly reduces them on the fiber surface without an external reducing agent. Their approach controls the size and surface density of AgNPs by modulating the reaction time and silver nitrate concentration. Notably, this process improves the antibacterial performance and reduces the silver-ion release rate of the composite without affecting the crystalline structure of the silk fibers (Lu et al., 2015).

## 4 Antimicrobial applications of PDA NPs

### 4.1 Direct antimicrobial properties

Polydopamine nanoparticles exhibit intrinsic antimicrobial activity mainly because the redox reactions between the catechol/quinone groups of PDA and molecular oxygen generate reactive oxygen species (ROS) (Jabbar et al., 2023; Smies et al., 2022; Soto-Garcia et al., 2023; Souza et al., 2025). Electron transfer in the catechol and quinone groups yields different ROS; for instance, catechol oxidation produces hydrogen peroxide (H_2_O_2_), electron transfer from PDA to molecular oxygen (O_2_·^–^) produces superoxide anions, and catechol oxidation forms hydroxyl radicals (·OH) and, rarely, singlet oxygen (^1^O_2_). Besides electron transfer, these reactions are sometimes attributed to ion isolation and the synergistic interaction of PDA with other materials (Kan et al., 2025; Kensel Rajeev et al., 2024; Smies et al., 2022; Souza et al., 2025; Wang J et al., 2024; X. Wang et al., 2025). The antibacterial action of PDA–ROS highly depends on the environmental conditions. For example, the pH of the medium influences the polymerization and hence the redox behavior of PDA. Alkaline environments accelerate the polymerization and favor the formation of semiquinone radicals (Souza et al., 2025), whereas acidic solutions tend to inhibit dopamine oxidation and reduce ROS formation (Lv et al., 2025). Overall, ROS causes oxidative damage to bacterial membranes, proteins, and nucleic acids, thus causing structural and functional disruption and ultimately inducing cell death (Cicogna et al., 2023; Jabbar et al., 2023; Smies et al., 2022; Soto-Garcia et al., 2023; Souza et al., 2025).

Furthermore, PDA can physically interact with the bacterial cell membrane, causing structural damage mainly through strong adhesion. This interaction can involve the chelation of essential ions or interaction with membrane proteins that are essential for bacterial function. In specific cases, *in situ* polymerization of PDA on bacterial surfaces can form a coating that suffocates cellular activity (Costa et al., 2024; Dou et al., 2024; Lv et al., 2025).

However, the reported direct antimicrobial activities of pure PDA NPs vary across studies, and antimicrobial action is influenced by the concentration and form of the nanoparticles, environmental conditions (such as pH and oxygen level), the species and structure of the bacteria, and the application of external stimuli. Most studies have concluded that pristine PDA lacks remarkable antimicrobial activity (Dou et al., 2024; Jabbar et al., 2023; Kensel Rajeev et al., 2024; Shumbula et al., 2022; Souza et al., 2025); however, PDA NPs combined with other antimicrobial strategies can contribute antimicrobial action through mechanisms such as photothermal/photodynamic enhancement, ROS generation, and membrane interaction, enhancing the antimicrobial efficacy of the overall system.

### 4.2 Drug delivery systems

With their strong adhesiveness, biocompatibility, chemical reactivity, and biodegradability (Bano et al., 2023; Patel et al., 2023), PDA NPs are promising drug delivery systems offering multiple drug-loading strategies. In one strategy, therapeutic agents are encapsulated within the porous structure of PDA. For example, the biocompatible antimicrobial compound curcumin has been effectively encapsulated into a PDA-coated zinc metal–organic framework (MOF), forming a drug delivery system with high bactericidal efficacy (Jabbar et al., 2023). Another method relies on surface adsorption. The surfaces of PDA NPs can be loaded with biomolecules through π−π stacking, hydrogen bonding, or electrostatic interactions. Finally, drugs can be covalently bonded to the functional groups on PDA, providing a stable drug-loading and release platform (Batul et al., 2023; Yu et al., 2022).

An illustrative case is the HPDA@GLA/AMP nanoplatform (HPDA = hollow PDA; GLA = glycyrrhizic acid; AMP = antimicrobial peptide), developed by Wang, Dong, and others (2024) for treating bacterial keratitis. Besides exemplifying the versatility of PDA as a nanocarrier, this system demonstrates engineering of PDA-based platforms to simultaneously inhibit bacterial infection and inflammatory damage. HPDA NPs are synthesized by coating silica NPs with PDA via autooxidation of dopamine, followed by etching of the silica core. The resulting hollow structure largely increases the drug-loading capacity. Moreover, HPDA retains the antioxidant and ROS-scavenging properties of PDA, providing an immunomodulatory function that contrasts with that of passive carriers. To achieve targeted delivery, the authors further functionalized HPDA with 3-aminophenylboronic acid and hyaluronic acid (HPBH), enabling dual-targeting functionality. Finally, the HPBH nanocarrier was loaded with two bioactive agents: AMPs and GLA. The platform showed high antibacterial efficacy against *Pseudomonas aeruginosa* and *S. aureus*, inhibiting more than 90% of bacterial growth at therapeutic concentrations *in vitro*. An *in vivo* study in a mouse model of bacterial keratitis confirmed the therapeutic efficacy of the HPBH@GLA/AMP system. Wang R et al. (2024) observed bacterial elimination, attenuated inflammation, and intensified wound healing.

In addition, the polymeric network of PDA facilitates controlled and stimuli-responsive drug release, enabling precise control over therapeutic delivery (Bano et al., 2023; Soto-Garcia et al., 2023). For instance, Yu et al. (2022) demonstrated that near-infrared light (NIR) increases levofloxacin (Levo) release from PDA@Levo NPs. NPs respond to the typical acidic pH levels of biofilms and elevated temperatures, rapidly delivering the drug to biofilms under NIR light. Supporting this study, Patel et al. (2023) highlighted the effectiveness of PDA NPs in stimuli-responsive drug delivery systems. They developed a nanocomposite hydrogel system incorporating PDA NPs and thermoresponsive liposomes loaded with antibiotics. Under an NIR laser, the PDA triggered sequential drug release from the liposomes. Many controlled delivery applications use PDA as a coating material that modulates the diffusion of loaded agents. For instance, in various systems designed for antibacterial silver delivery, PDA coatings immobilize silver NPs (AgNPs) on diverse substrates, reducing nanoparticle leakage and enabling gradual Ag^+^ ion release over extended periods of time (Batul et al., 2023; Lu et al., 2015).

### 4.3 Photothermal therapy

As confirmed in several studies, PDA inherently possess strong photothermal properties, especially under NIR light with a wavelength of 808 nm, stimulating rapid light conversion into heat (Lin et al., 2025; Lv et al., 2025; Soto-Garcia et al., 2023). This property arises from the structure of PLA. The electron donor–acceptor characteristics of the planar conjugated system enable light absorption over a broad spectrum, including the NIR region (N. Xu et al., 2023). The light energy adsorbed by PDA NPs excites the electrons within the PDA structure. Later, the excited electrons relax and release the absorbed energy as heat (Soto-Garcia et al., 2023). Ultimately, this process raises the local temperature to a level that can damage bacterial membranes (Lin et al., 2025; Lv et al., 2025; Soto-Garcia et al., 2023). NIR radiation easily penetrates tissue, allowing treatment of deeper infections (Z. P. Li et al., 2022). In addition, the generated heat can disrupt cellular metabolism, leading to bacterial death (Lv et al., 2025; Soto-Garcia et al., 2023). PDA nanomaterials exhibit excellent photothermal stability with no substantial efficiency loss after various heating–cooling cycles (Cai et al., 2024; T. Li et al., 2024; Lin et al., 2025; Liu et al., 2025; Qi et al., 2022; Wang J et al., 2024; X. Wang et al., 2025; Wu et al., 2023; N. Xu et al., 2023; Yang et al., 2025; Zeng et al., 2021; X. Zhao et al., 2023).

Several mechanisms drive the antimicrobial effects of heat generation. Elevated temperatures can disrupt bacterial cell membranes, ultimately causing cell lysis (T. Li et al., 2024; Liu et al., 2025). High temperatures also denature and inactivate proteins, nucleic acids, and enzymes within bacteria, thereby suppressing their biological functions. Moreover, photothermal therapy can disrupt the extracellular matrix of bacterial biofilms (T. Li et al., 2024; Wang J et al., 2024; Zeng et al., 2021; Zhao Y et al., 2024). Although elevated temperatures are highly effective antimicrobial treatments, excessively high temperatures can damage the surrounding healthy tissue (X. Zhao et al., 2023). Consequently, current strategies leveraging the photothermal capacity of PDA often rely on synergistic effects that operate at more biocompatible lower temperatures. These mechanisms remain effective when synergized with other mechanisms that increase cell-membrane permeability or promote catalytic activity (T. Li et al., 2024; Wu et al., 2023; N. Xu et al., 2023).

Moreover, the photothermal performance of PDA NPs depends on various additional factors. Increasing the concentration of PDA-containing materials is known to raise the local temperature under the same irradiation power (T. Li et al., 2024; Wang J et al., 2024; Wu et al., 2023; Yang et al., 2025; Zeng et al., 2021; Zhao Y et al., 2024). Similarly, increasing the power of the NIR laser considerably increases the temperature rise rate. The photothermal efficiency of PDA can be improved by incorporating PDA into other materials (T. Li et al., 2024; Qi et al., 2022).

### 4.4 Integration of PDA NPs with antimicrobial peptides

Antimicrobial peptides (AMPs) are short proteins with broad-spectrum inhibitory activity against various pathogens, including bacteria and fungi. Typical AMPs comprise 10–60 amino acid residues that play critical roles in the natural immune response. AMP molecules are characteristically amphipathic, possessing hydrophilic and hydrophobic areas, and are commonly cationic, facilitating their interaction with microbial membranes (Siddiqui et al., 2024; Talapko et al., 2022). This subsection discusses the combination of PDA NPs with AMPs or peptidomimetics. This innovative strategy is being explored as a bacterial infection treatment, particularly against antimicrobial-resistant organisms.

Owing to its inherent adhesive and surface-reactive properties, PDA is an ideal coating or nanocarrier candidate for immobilizing or encapsulating AMPs (Browne et al., 2022). PDA NPs integrated with AMPs synergistically enhance the therapeutic efficacy against a broad spectrum of bacterial strains, including multidrug-resistant strains. For instance, ZnO NPs, which are known to generate intracellular ROS and disrupt bacterial membranes, have been effectively coated with a PDA coupling interface that immobilizes AMP GL13K, a 13-amino-acid peptide with potent antimicrobial activity against *S. aureus* and *E. coli*. The resulting ZnO@PDA/GL13K nanoplatform considerably enhances the bactericidal activity from that of unmodified ZnO, even at low peptide concentrations (Zhang X et al., 2024). Similarly, PDA-coated ZnO NPs have been conjugated with the antimicrobial peptide ε-poly-L-lysine (ε-PL) to form a multifunctional nanocomposite in which ZnO serves as a broad-spectrum antimicrobial agent, PDA serves as a linker layer and performs photothermal conversion, while ε-PL naturally improves bacterial targeting through electrostatic interactions. This composite demonstrated high antibacterial efficacy, eliminating 99.70% of *E. coli* and 100% of *S. aureus* at low dosages (<100 μg/mL), highly outperforming its individual components. The performance improvement was attributed to a dual mechanism: ε-PL facilitates initial bacterial capture via electrostatic interactions, whereas PDA provides photothermal heating that disrupts the membrane, eventually leading to cell death. In addition, the platform exhibited strong biofilm disruption, eradicating established *S. aureus* biofilms (Qi et al., 2022).

Browne et al. (2022) applied PDA as a versatile linking agent in the functionalization of biomaterial surfaces. Their strategy uses three synthetic peptidomimetic AMPs—melimine, Mel4, and RK758—selected for their broad-spectrum efficacy against clinically relevant bacterial pathogens, including drug-resistant strains. The PDA-functionalized surfaces completely eradicated *S. aureus* and demonstrated substantial activity against Gram-negative bacteria such as *E. coli* and *P. aeruginosa*. The antimicrobial mechanism was inferred as interaction and disruption of the cell membrane, consistent with the amphipathic and cationic nature of peptidomimetics. More recently, Peng et al. (2025) synthesized a pH-responsive hydrogel coating comprising chitosan (CS) and PDA, embedded it with the AMP HHC36, and applied it to titanium substrates. They selected titanium because it is used in implantable medical devices, CS because it possesses intrinsic antimicrobial and pH-sensitive properties, PDA because it provides strong wet adhesion and can potentially destroy bacteria through a photothermal mechanism, and the synthetic AMP HHC36 because it effectively eliminates multidrug-resistant bacteria or superbugs. The resulting hydrogel was largely effective against *S. aureus* and *E. coli*, with inhibition rates above >90%. Remarkably, the pH-responsiveness of the hydrogel enabled the controlled release of HHC36 in the acidic microenvironments commonly found at bacterial infection sites, thus optimizing the therapeutic efficacy of the composite while minimizing off-target effects.

### 4.5 Fenton-like reaction improvement via PDA NPs: a synergistic approach

In therapeutic systems integrating PDA with Fenton-like catalysts, PDA with intrinsic photothermal properties induces localized hyperthermia under NIR irradiation, thus improving the catalytic activity of the Fenton-like agents. This synergistic mechanism notably increases the ROS production and strengthens the bactericidal effect (Gao et al., 2024; Zhao Q et al., 2024). Such synergy between the photothermal effect and chemodynamic therapy is commonly known as the photoenhanced Fenton effect or synergetic photothermal–chemodynamic therapy. In this context, PDA is often used as a multifunctional platform that incorporates or coordinates with transition metal ions that catalyze Fenton or Fenton-like reactions, increasing the therapeutic efficacy of the platform (N. Xu et al., 2023; Zhao Q et al., 2024).

In composites such as PDA@FeS, PDA stabilizes the material by promoting uniform dispersion and inhibiting the oxidation of FeS. Meanwhile, sulfur ions facilitate the reduction of ferric (Fe^3+^) to ferrous (Fe^2+^) iron, accelerating the Fenton-like conversion of H_2_O_2_ to hydroxyl radicals (·OH). This catalytic activity (a key mechanism in chemodynamic therapy) is further potentiated by the photothermal effect of PDA under NIR irradiation, enhancing ROS generation and therapeutic efficacy (N. Xu et al., 2023). Similarly, Wu et al. (2023) designed a copper-based composite with PDA and FDM-23, a 2D metal-organic framework that amplifies peroxidase-like activity to promote antibacterial effects. This composite catalyzes the decomposition of H_2_O_2_ to toxic OH radicals while depleting intracellular glutathione and weakening the antioxidant defense mechanisms of bacteria. The resulting increase in cuprous ion (Cu^+^) levels further amplifies ROS production by improving the efficiency of redox cycling, ultimately contributing to effective bacterial eradication.

Another interesting example is the cuprous oxide (Cu_2_O)–tin oxide (SnO_2_)-doped PDA (CSPDA) composite, which integrates a heterostructured nanoenzyme core with a polydopamine shell to achieve synergistic Fenton-like catalytic and photothermal effects. In this design, Cu_2_O nanocubes are first synthesized and then coated with SnO_2_ NPs. The P-type Cu_2_O and N-type SnO_2_ semiconductros possess similar lattice parameters, facilitating the formation of a P–N junction that improves the interfacial charge transfer and catalytic efficiency. This heterostructure catalyzes the decomposition of H_2_O_2_ into hydroxyl radicals (·OH) through a Fenton-like mechanism. Unlike traditional Fenton systems, which typically require weakly acidic conditions, the Cu^+^/Cu^2+^ redox cycle in Cu_2_O achieves effective catalysis over a wide pH range encompassing weakly acidic, neutral, and weakly basic environments. The PDA coating plays triplicate roles as a biocompatible encapsulating matrix, an enhancer of the photothermal response under NIR irradiation, and an additional ROS generator via localized hyperthermia. This combination of chemodynamic and photothermal functionalities enhances the overall catalytic and antibacterial performance of the nanoplatform. CSPDA demonstrated effective peroxidase activity and a high photocatalytic potential for antibacterial therapy and oxidative stress–mediated treatments (Gao et al., 2024).

### 4.6 PDA NPs for the eradication of established drug-resistant bacterial biofilms

**FIGURE 3:**
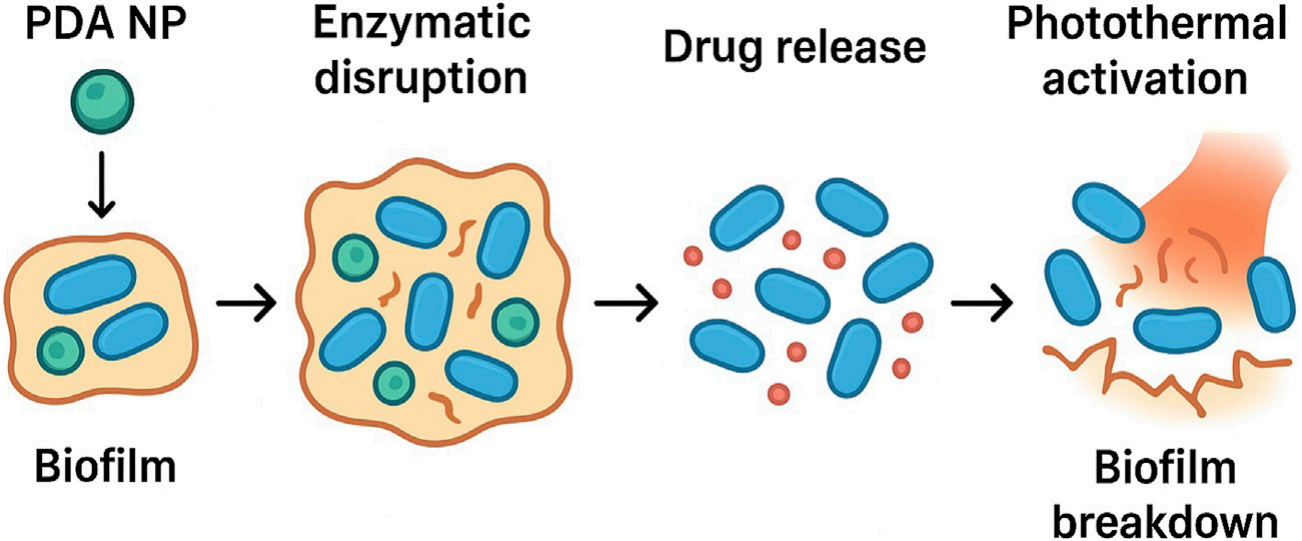
Schematic representation of PDA nanoparticle penetration and biofilm disruption.

The emergence of multidrug-resistant bacteria is a major public health concern, exacerbated by biofilm formation. Biofilms are dynamic bacterial communities embedded in a protective self-produced extracellular polymeric substance (EPS) matrix that impedes antibiotic penetration, reduces drug efficacy, and promotes persistent infections (Lv et al., 2025; Wang J et al., 2024). PDA-based materials can potentially eradicate mature bacterial biofilms through synergistic mechanisms involving enzymatic disruption, antibiotic delivery, and photothermal activation, often exploiting the adhesive capabilities of PDA as shown in Figure 3. This section highlights recent strategies using PDA NPs for eradicating bacterial biofilms.

Yu et al. (2022) combined enzymatic degradation, antibiotic therapy, and the photothermal effect into a dissolving microneedle patch. The enzyme $\left(\alpha \right.$ -amylase) degrades the extracellular polysaccharide matrix of a biofilm, exposing the bacteria. Simultaneously, the microneedle dissolves and releases Levo-loaded PDA NPs. Next, mild photothermal heat ( ∼ 50 °C) is generated under an NIR laser. The patch delivered promising results, reducing the biofilm masses of *S. aureus* and *P. aeruginosa in vitro* and *in vivo*. Remarkably, the α-amylase–PDA@Levo microneedles more effectively degraded *S. aureus* biofilms than *P. aeruginosa* biofilms, possibly reflecting the different polysaccharide matrices of the two species. After 24 h of incubation with the microneedles and NIR irradiation, only 12.63% ± 1.86% of the *S. aureus* biofilm biomass remained on the surface of the 48-well plate (Yu et al., 2022).

**FIGURE 4:**
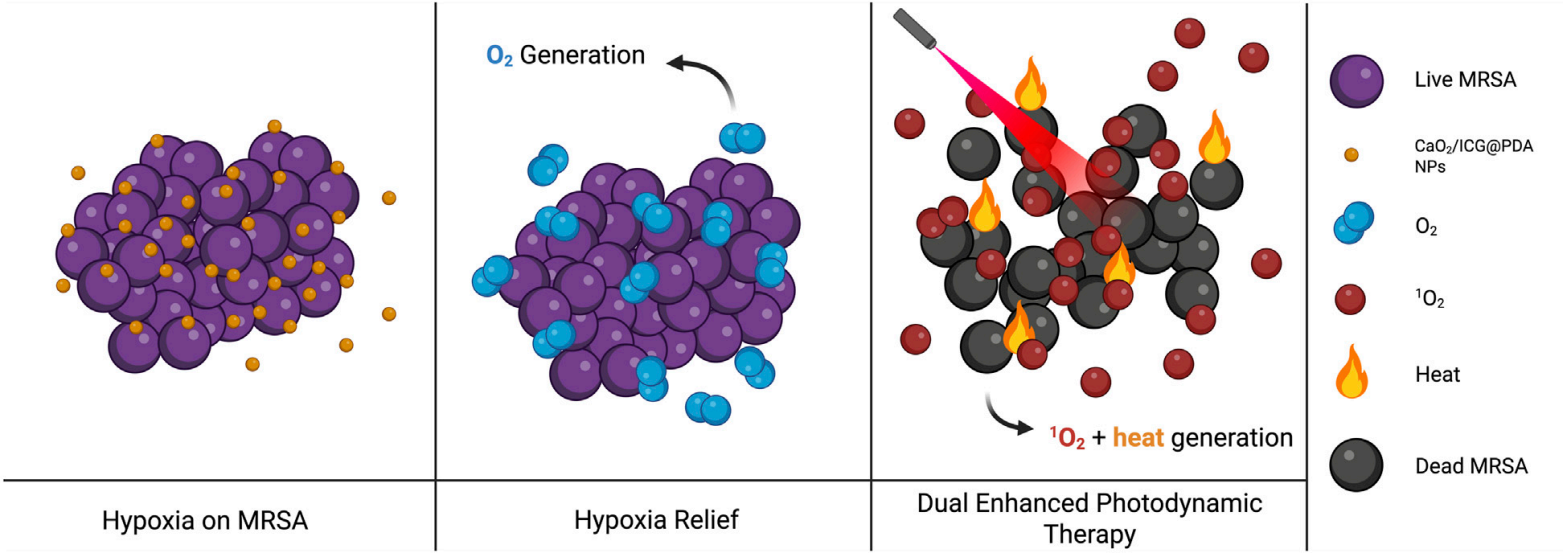
Schematic illustration of the dual-functional CaO_2_/ICG@PDA system improving PDT outcomes against MRSA biofilms. Image inspired on (Zhang H et al., 2024) (image prepared using BioRender.com).

Zhang R et al. (2024) designed a PDA with enhanced antibiofilm efficacy for use in photodynamic therapy platforms. Their CaO_2_/ICG@PDA system (ICG = indocyanine green) generates oxygen to alleviate hypoxia in an acidic biofilm environment. Under NIR activation, ROS generation by ICG enhances the efficacy of photodynamic therapy and PDA heats the local area. This system increases membrane permeability and bacterial mortality, eliminating over 99.9999% of methicillin-resistant *S. aureus (MRSA) in vitro* as shown in Figure 4 (Zhang H et al., 2024).

Another approach polymerizes dopamine *in situ* within a preformed biofilm. The dopamine monomer of PDA is allowed to diffuse throughout the biofilm structure. Ferrous ions are then externally added to catalyze the polymerization process. The reaction proceeds even under acidic conditions, thus forming PDA within the biofilm. Besides physically restricting microbial movement and activity, this mechanism prepares the system for subsequent photothermal therapy. PDA strongly converts NIR irradiation to heat, causing localized hyperthermia that damages the EPS structure and compromises bacterial membranes. This dual-action mechanism effectively disrupts the biofilm integrity from within, offering a controllable and targeted approach with minimal systemic toxicity (Lv et al., 2025).

In a more complex strategy designed by Wang J et al. (2024), Fe_3_O_4_@AgAu@PDA nanospindles are engineered to exploit magnetic and photothermal modes for enhanced biofilm elimination. These nanoparticles comprise a magnetic Fe_3_O_4_ core, a layer of AgAu nanorods, and an outer PDA shell with high biocompatibility and photothermal sensitivity. The spindle-like morphology deeply penetrates the biofilm matrix, whereas the Fe_3_O_4_ core is externally controlled by a rotating magnetic field that induces rotation and mechanical agitation of the nanospindles, enabling effective disruption of the biofilm structure. Meanwhile, NIR irradiation activates heat production by both AgAu and PDA, accelerating bacterial death. This multimodal attack—mechanical disruption, localized hyperthermia, and adverse chemical effects (Ag^+^ release and ROS generation)—is remarkably more efficacious than conventional therapies (Wang J et al., 2024).

### 4.7 *In vivo* evaluation of PDA NPs for antimicrobial applications

In the past few years, the antimicrobial properties of PDA NPs against diverse bacterial strains have been studied *in vivo*. These studies have increased our understanding of antimicrobial efficacy under physiological conditions and have revealed the biocompatibility, biodistribution, and potential toxicity of nanomaterials. This section summarizes representative *in vivo* studies, highlighting the key findings supporting the use of PDA NPs in next-generation antimicrobial therapies.

The delivery and *in situ* polymerization of dopamine have been investigated in a rat model of severe biofilm-associated wound infection. Lv et al. (2025) created full-thickness skin defects in rats and inoculated them with *P. aeruginosa*, a pathogen recognized for its biofilm-forming capacity and resistance to antimicrobial treatment. The infected wounds were subjected to different treatments: phosphate-buffered saline as a negative control, NIR light irradiation alone, *in situ* polymerized PDA, and combined PDA and NIR irradiation. Over a 14-day treatment period, the PDA–NIR combination greatly reduced the bacterial burden and effectively disrupted the biofilm matrix, removing biofilm layers as thick as 120 μm; by contrast, the control, PDA, and NIR-alone groups exhibited minimal antibacterial activity. Histological analysis revealed accelerated wound healing and reduced inflammatory cell infiltration after the PDA-based treatment, indicating antimicrobial efficacy and enhanced tissue regeneration. Furthermore, biosafety assessments based on hemolysis assays showed no remarkable cytotoxic effects at concentrations up to 400 μg/mL, indicating the therapeutic potential and biocompatibility of the system (Lv et al., 2025).

PDA NPs modified with cationic polymers have demonstrated similar potential as the promoters of bacterial wound healing. Cai et al. (2024) engineered an alternating polymeric coating to enhance the penetration of PDA NPs into dense bacterial biofilms. Under NIR irradiation, the photothermal effect induced by PDA disrupts the coating–NP interactions, triggering the controlled and direct release of the cationic antimicrobial polymer into the bacteria. To test the therapeutic efficiency of this *in situ* antimicrobial delivery for targeted bacterial eradication, the researchers infected a mouse wound model with methicillin-resistant *S. aureus* and treated it with seven different formulations. The group receiving NIR-irradiated polymer-functionalized PDA NPs exhibited the best therapeutic outcome, with a large reduction in wound size within the first 24 h and near-complete healing within 7 days. Histological analyses, including hematoxylin/eosin (H&E) staining and immunohistochemistry on days 3 and 7, confirmed a remarkable reduction in inflammatory cell infiltration and enhanced tissue regeneration. *In vivo* biosafety assessments, including body weight monitoring, histological examination of major organs, and serum biochemical and hematological analyses, revealed no remarkable abnormalities, indicating the high biocompatibility and safety of the treatment.

**FIGURE 5:**
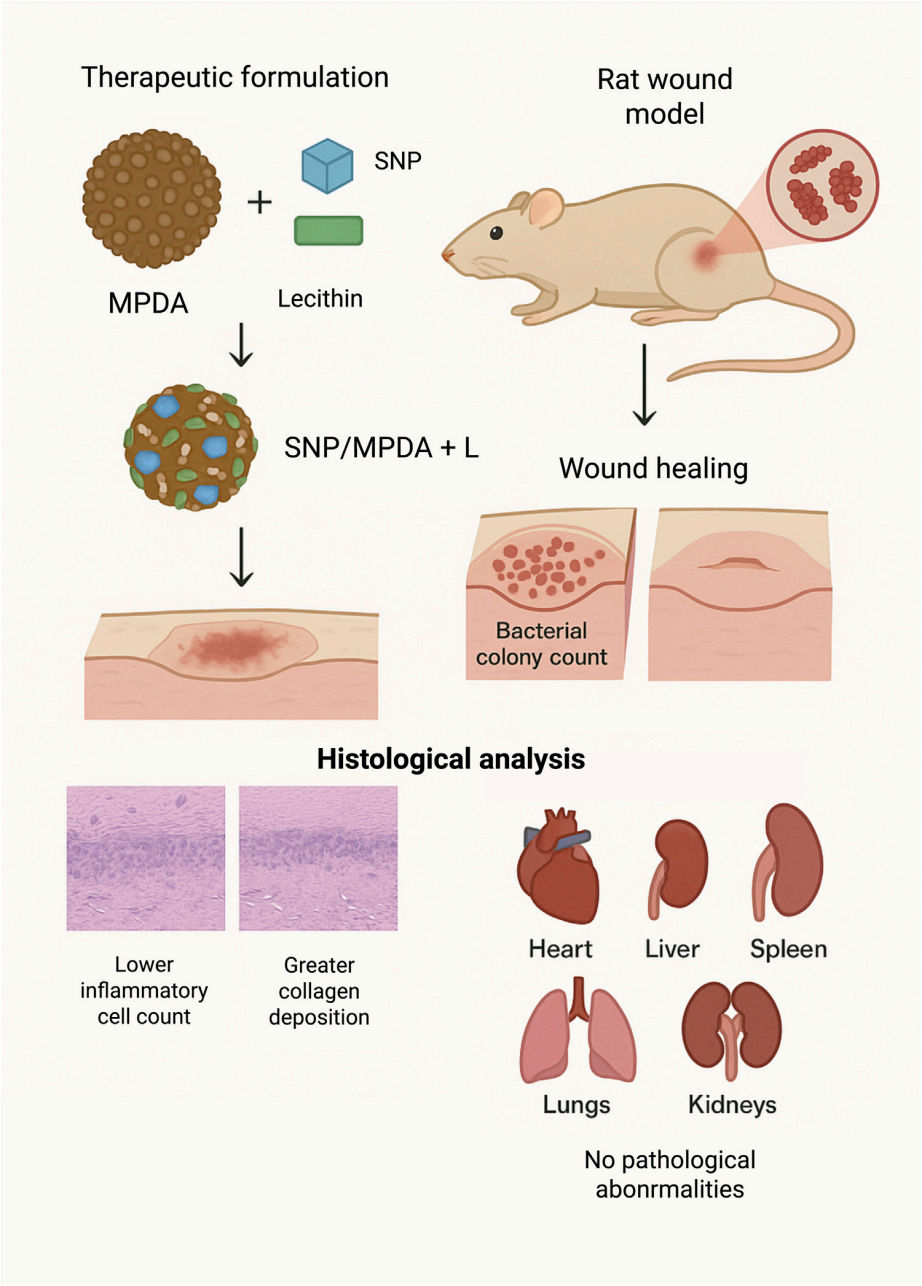
Synergistic Antibacterial and Wound-Healing Effects of SNP/MPDA + L Nanoplatform under NIR Irradiation in an S. aureus–Infected Rat Model (image prepared using BioRender.com).

In another recent study, Wang, Ma, and others (2024) functionalized a mesoporous PDA nanocarrier with sodium nitroprusside (SNP) and lecithin (SNP/MPDA + L) for synergistic antibacterial activity and wound-healing promotion. The photothermal properties of MPDA, together with the nitric oxide–releasing capability of SNP, enhanced the therapeutic efficacy. The therapeutic formulation combined with NIR irradiation was administered to an *S. aureus*–infected rat wound model over a 9-day period. The SNP/MPDA + L + NIR treatment considerably reduced the bacterial colony count from that of the control group and accelerated wound healing. Histological analysis confirmed a marked reduction in the inflammatory cell count and greater collagen deposition at the wound site, indicating a higher degree of tissue regeneration than in the control group. Furthermore, the histological examination of the major organs (heart, liver, spleen, lungs, and kidneys) revealed no pathological abnormalities, suggesting the biocompatibility and safety of the nanotherapeutic platform as shown in Figure 5.

Alternatively, Xu et al. (2025) functionalized poly (lactic-*co*-glycolic acid) with PDA and polyethylene glycol–thiol (PEG–SH) (PEG–PDA@PLGA) microspheres as an oral formulation against intestinal bacterial infections. This multifunctional platform exploits the iron-chelating capability of PDA to coordinate iron ions while codelivering rifampicin and FeCl_3_. The system promotes localized ROS-generating Fenton reactions that efficiently eradicate pathogenic bacteria. The therapeutic efficacy was assessed in a mouse model infected with *Salmonella typhimurium*. *In vitro* assays demonstrated a remarkable 99% bacterial clearance within just 4 hours. *In vivo*, the PEG–PDA@PLGA treatment group achieved a survival rate of 100%, considerably outperforming the control group. Histological analyses (H&E staining) of treated tissues revealed lower inflammatory cell infiltration, less oxidative stress, and lower proinflammatory-factor expression in the treatment group than in the control group. Furthermore, biocompatibility assessments indicated minimal hemolytic activity (less than 5%) and no remarkable major pathological alterations within 48 h after administration, implying that PEG–PDA@PLGA microspheres are safe and effective antimicrobial therapies.

## 5 Conclusion: research gaps and future directions

Polydopamine (PDA) nanoparticles have emerged as versatile and promising smart antimicrobial agents owing to their intrinsic biocompatibility, tunable surface chemistry, and robust photothermal properties. Recent advancements have improved synthesis methods, including solution oxidation, electropolymerization, and enzymatic catalysis, that enable control over particle size, morphology, and coating thickness. Diverse functionalization strategies such as metal-ion coordination, polymer grafting, and AMP incorporation have further endowed PDA nanomaterials with synergistic antibacterial, antibiofilm, and stimuli-responsive drug delivery capabilities. These innovations have successfully broadened the antimicrobial and biomedical applications of PDA-based platforms, ranging from biofilm eradication and wound healing to targeted therapies for drug-resistant infections (Table 1 provides an overview of the articles discussed in this review).

**TABLE 1:** Overview of polydopamine (PDA)-based strategies against antimicrobial resistance.

PDA nanostructure	Functionalization	Morphology	Role of PDA	Application	References
PDA nanoparticles (NPs)	Levofloxacin (Levo)	Spherical PDA@Levo NPs	Photothermal agent, antioxidant, and cell adhesion, controlled drug loading	Triple therapy against bacteria and biofilms	Yu et al. (2022)
Hollow PDA (HPDA) NPs	3-aminophenylboric acid, hyaluronic acid, antimicrobial peptide (AMP), and glycyrrhizic acid (GLA)	Hollow HPBH@GLA/AMP nanoplatform	Nanocarrier and ROS-scavenger	Dual-targeted antibacterial and cascaded immunomodulatory therapy for bacterial keratitis	Wang R et al. (2024)
PDA layer	ZnO nanoparticles (NPs), GL13K peptide	ZnO@PDA/GL13K hybrid	Covalent bonding agent attaching GL13K to ZnO NP surfaces and facilitates grafting	Treating bacterial infected wounds and enhancing antimicrobial performance	Zhang X et al. (2024)
PDA coating	Antimicrobial peptidomimetics (melimine, Mel4, RK758)	Coated biomaterial surfaces	Versatile linking agent	Prevention and treatment of biofilm-mediated infections in implantable medical devices	Browne et al. (2022)
PDA coating	Polypropylene (PP) nonwoven fabric (NWF)	Coated PP NWF	Adherent, antioxidant, and antimicrobial properties	Enhancing the functionality of PP NWF in face masks for protection against viruses and bacteria	Zhao Q et al. (2024)
PDA (part of hydrogel)	Chitosan, HHC36 (antimicrobial peptide)	Hydrogel coatings on titanium surfaces	Provides wet adhesion, antimicrobial activity, and free radical scavenging	pH-responsive hydrogel coatings with antibacterial properties against MRSA	Peng et al. (2025)
PDA layer	Dhvar5 (AMP), MSI78 (AMP)	AMP-coated titanium (AMP-Ti) substrates	Grafting agent and long-term stability	Prevention of orthopedic device-related Infections and bactericidal agent against MRSA	Costa et al. (2024)
*In situ* polymerized dopamine	Ferrous ions (Fe^2+)^	PDA coating on EPS and bacteria in biofilms	*In situ* polymerization to penetrate biofilms via photothermal effect	Efficient penetration and eradication of bacterial biofilms	Lv et al. (2025)
PDA coating	ZnO nanoparticles, ε-poly-L-lysine (ε-PL) (AMP)	ZnO@PDA-ε-PL nanohybrid (short rod-shaped)	Photothermal agent facilitates the conjugation of ε-PL	Photothermal antibacterial agents for synergistic killing and biofilm eradication	Qi et al. (2022)
PDA NPs	Synthetic cationic antimicrobial polymers (PS+, P(S + OH), AMP-mimicking)	Polymerized PDA NPs	Photothermal agent as a base for polymer loading, provides high local charge density for adhesion, and photothermal release	Targeted bacterial killing (MRSA, *E. coli*), enhanced biofilm penetration, and MRSA-induced wound healing	Cai et al. (2024)
PDA submicron spheres	Silver nanoparticles (AgNPs)	Submicron particles on gauze	Reducing and stabilizing agent for AgNPs, provides strong cell adhesion, binds AgNPs to gauze, and releases AgNPs	Enhances antimicrobial performance in medical gauze and inhibits biofilm formation	Cui et al. (2024)
PDAsubmicron particles	AgNPs	Coating titanium alloys	Loads AgNPs, acts as a reducing and stabilizing agent, strong cell adhesion, and multilayer coating	Prevents bacterial adhesion and colonization on implant surfaces and prevents biofilm formation	Cui et al. (2025)
PDA-coated substrates	Ag NPs and gentamicin (an antibiotic)	Coatings on glass slides	Supports Ag NP immobilization, gentamicin loading, adhesive, and reductive properties	Prevents bacterial colonization and provides effective long-term antimicrobial properties	Batul et al. (2023)
PDA binding layer, surface coating, and thin film	Green-synthesized AgNPs	Multilayered coating on urinary catheters	Anchors antibacterial agents to biomaterials, acts as a crosslinker, and provides durability and stability through self-polymerization	Prevents multidrug-resistant *P. mirabilis* infection and urinary catheter incrustation; reduces biofilm formation	Saikawa et al. (2024)
PDA coating	Chitosan and AgNPs combined with laser-induced periodic surface structures on titanium	Composite coating	Hydrophilic coating, biocompatibility, and stability, supports biopolymer grafting, reduces metal NPs	Hybrid antibacterial surfaces, superior resistance to biofilm formation, and stable antifouling performance	Wang Y et al. (2024)
PDA-coated hydroxyapatite (HA)	HA and polyvinyl alcohol (PVA)	Nanocomposite films	Prevents HA agglomeration, strongly binds to PVA, improves mechanical performance, provides strong interfacial adhesion, mineralization, and HA accumulation	High antibacterial capacity against *A. Baumannii*, *S. mutans*, and MRSA	Erdem et al. (2022)
PDA NPs	Zeolite imidazolate framework-8 (ZIF-8) and Ag particles	Nanoparticles (ZIF-8/Ag/PDA)	Extends the ZIF-8 light therapy range to NIR, enhances photothermal conversion efficiency, improves the stability of ZIF-8, and enhances photothermal sterilization	Combats misuse of antibiotics (*S. aureus*, *E. coli*), promotes liberation of metal ions (Ag^+^, Zn^2+^), and can potentially replace antibiotics	Lin et al. (2025)
PDA-modified nanosheets	2D FDM-23 Metal–organic framework	Composite nanosheets	Photothermal effect enhances peroxidase-like catalytic activity, generates hydroxyl radicals, and contributes to glutathione depletion	Combats drug-resistant bacteria (*S. aureus*, *E. coli*) and offers synergistic antibacterial therapy (photothermal-enhanced chemodynamics)	Wu et al. (2023)
PDA NPs	AuAg bimetallic nanoparticles and MoS_2_-based composite nanomaterials	Composite nanomaterials	Facilitates composite formation via adhesion	Skin-wound healing for drug-resistant bacterial infections and exerts antimicrobial and anti-inflammatory effects	Yang et al. (2025)
PDA thin photothermal layer/coating	Nanopillar surface (mechano-bactericidal)	Nanostructured surface with PDA coating	Photothermal agents with a nanopillar surface, biomimetic polymer, adhesion, and biocompatibility and increase the susceptibility of bacterial cell membranes to structural rupture	Enhanced antibacterial efficiency against *S. aureus* and *E. coli*; prevents drug resistance through physical mechanisms	X. Zhao et al. (2023)
Mussel-like PDA/wrapper/nanomaterials	Aggregation-induced emission nanodots (tetraphenylethylene (TPE) molecules)	Nanodot (TPE@PDA Nanodot)	Adheres efficiently to the bacterial-membrane surface, enhances bioavailability, and provides intrinsic antimicrobial effect	Broad-spectrum antimicrobial activity (Gram-positive/negative, drug-resistant MRSA) and accelerates wound healing	Dou et al. (2024)
PDA nanocoating	Cuprous oxide (Cu_2_O)	Coating adhesive-backed polyethylene wraps	Adhesive and polymerizes on most solid materials	Bactericidal activity against *P. aeruginosa*	Behzadinasab et al. (2023)
PDA coating	Silver nanozymes (PDA/PM-AgNPs) crosslinked with cationic guar gum (CG)	Hydrogel (CFC-PDA/Ag)	Forms coating for silver nanozymes and contributes to notable photothermal conversion efficiency	Manages wounds infected with multidrug-resistant bacteria, accelerates wound healing, and exhibits free radical scavenging capabilities	X. Wang et al. (2025)
PDA NPs	Cellulose nanocrystals (CNCs) modified with polyethyleneimine	Nanoparticles incorporated into cellulose acetate membranes	Surface chemical modifier of CNCs, which are a part of a mixed-charge system, and increases membrane surface roughness	Endotoxin scavenging and antibacterial activity for hemodialysis and blood purification	Zhou et al. (2024)
PDA NPs	Gold nanorods (AuNRs) and AgNPs	Nanocomposites (AgNPs decorated on AuNRs)	Improves the biocompatibility of AuNRs, modifies the underlying surface, and functions as an adhesive platform	Combats bacterial infections (*E. coli*, *S. aureus*) and combines photothermal and chemical bactericidal activity	Zhao Y et al. (2024)
PDA NPs	Thermally responsive liposomal nanocarriers, agarose hydrogel, antibiotic (rifampicin), and EDTA	Nanocomposite hydrogel	Highly efficient photoactive agents and carriers of drug molecules	On-demand laser-responsive antimicrobial delivery, prevents bacterial growth, and treats drug-resistant bacterial infections	Patel et al. (2023)
Functionalized PDA	AgNPs on silk fibers	Silk fiber coating	Biocompatible mimic, chelates metal ions, contributes redox activities, adheres to surfaces, serves as a 3D matrix for high-density AgNP loading, and slows the Ag^+^ release	Antibacterial agent used in the clothing and textile industry (*E. coli*, *S. aureus*) and exerts long-term antibacterial effects	Lu et al. (2015)
PDA NPs	Ferrous sulfide (FeS)	Nanoparticles (PDA@FeS NPs)	Prevents oxidation and agglomeration of FeS, synergistically enhances the photothermal effect, provides hydrophilicity and biocompatibility, coordinates metal ions, and assists dispersion	Effective against drug-resistant bacteria, demonstrates potent photothermal antibacterial activity (photothermal-enhanced Fenton reaction)	N. Xu et al. (2023)
PDA shell	Magnetic Fe_3_O_4_ nanospindle core and AgAu nanorods	Core-shell nanospindle	Highly biocompatible, bio-adheres to bacterial surfaces, provides good photothermal conversion efficiency, and acts as a coating layer	Combats drug-resistant bacteria, removes biofilm through a coupled force–thermal antibacterial effect, and offers long-term antibacterial function	Wang J et al. (2024)
PDA-coated TiO_2_ NPs (PDA PMO)	Titanium oxide and hyaluronic acid–dopamine hydrogels	Nanoparticles	Photocatalytic/photothermal, biomimetic, increases temperature to degrade bacterial membranes; is involved in electrostatic interactions, protein chelation, and ROS production	Prevents and treats bacterial infections (*E. coli*, *S. aureus*), prevents antibiotic resistance, and shows potential for wound healing and catheter infection prevention	Soto-Garcia et al. (2023)

Despite these advances, several challenges restrict the clinical translation of PDA nanomaterials. Biocompatibility assessments are often confused by interference in conventional cytotoxicity assays, and incomplete understanding of long-term biodegradation or potential off-target effects raises safety concerns, particularly in applications involving photothermal or combined therapies. Furthermore, limited standardization in evaluating antimicrobial efficacy and insufficient *in vivo* validation hinder the comparability and reproducibility of many reported results.

To fully realize the potential of PDA-based antimicrobial systems, future research should focus on establishing robust, reproducible synthesis protocols and standardized characterization methodologies that ensure predictable nanoparticle performance. Broad and rigorous evaluations of biocompatibility, biodegradability, and biosafety, including new assay designs and long-term *in vivo* studies, are crucial for advancing clinical translation. Additionally, interdisciplinary approaches integrating materials science, microbiology, and biomedical engineering will be essential for optimizing functionalization strategies, deciphering antibacterial mechanisms, and developing scalable, clinically viable PDA nanoplatforms.

Through coordinated efforts to address these prevalent weaknesses, PDA nanomaterials are well positioned to shape the next-generation of smart antimicrobial technologies, offering innovative solutions to combat the growing threat of antimicrobial resistance.

## References

[B1] Aflori M. (2021). Smart nanomaterials for biomedical applications—A review. Nanomaterials 11 (2). 396. 10.3390/nano11020396 33557177 PMC7913901

[B2] Almeida L. C. Frade T. Correia R. D. Niu Y. Jin G. Correia J. P. (2021). Electrosynthesis of polydopamine-ethanolamine films for the development of immunosensing interfaces. Sci. Rep. 11 (1), 2237. 10.1038/s41598-021-81816-1 33500469 PMC7838280

[B3] Bano S. Hasnain M. Rehman K. Kanwal T. Perveen S. Yasmeen S. (2023). Comparative analysis of polydopamine and casein coated Arabic gum stabilized silver nanoparticles for enhanced antimicrobial activity of quercitrin. J. Mol. Struct. 1294, 136515. 10.1016/j.molstruc.2023.136515

[B4] Battaglini M. Emanet M. Carmignani A. Ciofani G. (2024). “Polydopamine-based nanostructures: a new generation of versatile, multi-tasking, and smart theranostic tools,”, 55. Elsevier B.V. 10.1016/j.nantod.2024.102151

[B5] Batul R. Bhave M. Yu A. (2023). Investigation of antimicrobial effects of polydopamine-based composite coatings. Molecules 28 (11), 4258. 10.3390/molecules28114258 37298735 PMC10254676

[B6] Behzadinasab S. Williams M. D. Falkinham J. O. Ducker W. A. (2023). Facile implementation of antimicrobial coatings through adhesive films (Wraps) demonstrated with cuprous oxide coatings. Antibiotics 12 (5), 920. 10.3390/antibiotics12050920 37237824 PMC10215917

[B7] Browne K. Kuppusamy R. Chen R. Willcox M. D. P. Walsh W. R. Black D. S. (2022). Bioinspired polydopamine coatings facilitate attachment of antimicrobial peptidomimetics with broad-spectrum antibacterial activity. Int. J. Mol. Sci. 23 (6), 2952. 10.3390/ijms23062952 35328373 PMC8948759

[B8] Cai S. Hao Y. Wang X. Hu Y. Zhao J. Amier Y. (2024). Photothermal-mediated *in situ* delivery of polycations into bacteria *via* alternating polymer-modified nanoparticles for targeted bacterial killing and enhanced biofilm penetration. Adv. Funct. Mater. 35, 2413036. 10.1002/adfm.202413036

[B9] Caniglia G. Teuber A. Barth H. Mizaikoff B. Kranz C. (2023). Atomic force and infrared spectroscopic studies on the role of surface charge for the anti-biofouling properties of polydopamine films. Anal. Bioanal. Chem. 415 (11), 2059–2070. 10.1007/s00216-022-04431-7 36434170 PMC10079710

[B10] Caniglia G. Valavanis D. Tezcan G. Magiera J. Barth H. Bansmann J. (2024). Antimicrobial effects of silver nanoparticle-microspots on the mechanical properties of single bacteria. Analyst 149 (9), 2637–2646. 10.1039/d4an00174e 38529543

[B11] Chinemerem Nwobodo D. Ugwu M. C. Oliseloke Anie C. Al-Ouqaili M. T. S. Chinedu Ikem J. Victor Chigozie U. (2022). Antibiotic resistance: the challenges and some emerging strategies for tackling a global menace. John Wiley Sons Inc 36 (Issue 9), e24655. 10.1002/jcla.24655 35949048 PMC9459344

[B12] Cicogna F. Passaglia E. Elainaoui E. Bramanti E. Oberhauser W. Casini B. (2023). Coating of polypropylene non-woven fabric with layered double hydroxides bearing antioxidant and antibacterial natural compounds. Macromol. Chem. Phys. 224 (23), 2300148. 10.1002/macp.202300148

[B13] Costa B. Coelho J. Silva V. Shahrour H. Costa N. A. Ribeiro A. R. (2024). Dhvar5-and MSI78-coated titanium are bactericidal against methicillin-resistant *Staphylococcus aureus*, immunomodulatory and osteogenic. Acta Biomater. 191, 98–112. 10.1016/j.actbio.2024.11.016 39542199

[B14] Cui J. Shu H. Zhu P. Cao Z. Wang S. Cao P. (2024). Enhancing antimicrobial performance of gauze *via* modification by Ag-Loaded polydopamine submicron particles. J. Funct. Biomaterials 15 (6), 152. 10.3390/jfb15060152 38921526 PMC11205189

[B15] Cui J. Shu H. Gu X. Wu S. Liu X. Cao P. (2025). Enhancing antibacterial performance and stability of implant materials through surface modification with polydopamine/silver nanoparticles. Colloids Surfaces B Biointerfaces 245, 114327. 10.1016/j.colsurfb.2024.114327 39427395

[B16] Dou L. Wang X. Bai Y. Li Q. Luo L. Yu W. (2024). Mussel-like polydopamine-assisted aggregation-induced emission nanodot for enhanced broad-spectrum antimicrobial activity: *in vitro* and *in vivo* validation. Int. J. Biol. Macromol. 282, 136762. 10.1016/j.ijbiomac.2024.136762 39486741

[B17] D’Ischia M. Napolitano A. Ball V. Chen C. T. Buehler M. J. (2014). Polydopamine and eumelanin: from structure-property relationships to a unified tailoring strategy. Accounts Chem. Res. 47 (12), 3541–3550. 10.1021/AR500273Y 25340503

[B18] Erdem U. Dogan D. Bozer B. M. Turkoz M. B. Yıldırım G. Metin A. U. (2022). Fabrication of mechanically advanced polydopamine decorated hydroxyapatite/polyvinyl alcohol bio-composite for biomedical applications: *in-vitro* physicochemical and biological evaluation. J. Mech. Behav. Biomed. Mater. 136, 105517. 10.1016/j.jmbbm.2022.105517 36270152

[B19] Gao J. Yan Y. Gao S. Li H. Lin X. Cheng J. (2024). Heterogeneous Cu2O-SnO2 doped polydopamine fenton-like nanoenzymes for synergetic photothermal-chemodynamic antibacterial application. Acta Biomater. 173, 420–431. 10.1016/j.actbio.2023.11.009 37979634

[B20] GhavamiNejad A. Aguilar L. E. Ambade R. B. Lee S. H. Park C. H. Kim C. S. (2015). Immobilization of silver nanoparticles on electropolymerized polydopamine films for metal implant applications. Colloids Interface Sci. Commun. 6, 5–8. 10.1016/j.colcom.2015.08.001

[B21] Gholami Derami H. Gupta P. Weng K. C. Seth A. Gupta R. Silva J. R. (2021). Reversible photothermal modulation of electrical activity of excitable cells using polydopamine nanoparticles. Adv. Mater. 33 (32), 2008809. 10.1002/adma.202008809 34216406 PMC8363531

[B22] Hall W. McDonnell A. O’Neill J. (2018). Superbugs: an arms race against bacteria. Harvard University Press.

[B94] He G. Wan M. Wang Z. Zhou X. Zhao Y. Sun L. (2023). A simple surface modification method to prepare versatile PVDF electrospun nanofibrous felts for separation, sterilization and degradation. Prog. Org. Coat 182. 10.1016/j.porgcoat.2023.107664

[B23] Hemmatpour H. Haddadi-Asl V. Burgers T. C. Q. Yan F. Stuart M. C. A. Reker-Smit C. (2023). Temperature-responsive and biocompatible nanocarriers based on clay nanotubes for controlled anti-cancer drug release. Nanoscale 15 (5), 2402–2416. 10.1039/d2nr06801j 36651239

[B24] Husen A. Siddiqi K. S. (2023). Advances in smart nanomaterials and their applications. Elsevier, 23–50.

[B25] Jabbar A. Rehman K. Jabri T. Kanwal T. Perveen S. Rashid M. A. (2023). Improving curcumin bactericidal potential against multi-drug resistant bacteria *via* its loading in polydopamine coated zinc-based metal–organic frameworks. Drug Deliv. 30 (1), 2159587. 10.1080/10717544.2022.2159587 36718806 PMC9891165

[B26] Kan Z. Chen Y. Zhang Q. Pan L. Chen A. Wang D. (2025). Covalent bonding coating of quantum-sized TiO2 with polydopamine on catheter surface for synergistically enhanced antimicrobial and anticoagulant performances. Colloids Surfaces B Biointerfaces 245, 114249. 10.1016/j.colsurfb.2024.114249 39303386

[B27] Kensel Rajeev A. Sathish N. Elango H. Sivagnanam S. Nayak S. Das P. (2024). *In-situ* eco-friendly synthesis of a biomimetic robust antibacterial nanohybrid *via* catecholamine-induced metallization. Inorg. Chem. Commun. 170, 113172. 10.1016/j.inoche.2024.113172

[B28] Larrieu M. Mouniee D. Agusti G. Blaha D. Edouard D. (2024). Antimicrobial foam-filter based on commercial support coated with polydopamine and silver nanoparticles for water treatment. Environ. Technol. Innovation 33, 103468. 10.1016/j.eti.2023.103468

[B29] Li F. Yu Y. Wang Q. Yuan J. Wang P. Fan X. (2018). Polymerization of dopamine catalyzed by laccase: Comparison of enzymatic and conventional methods. Enzyme Microb. Technol. 119, 58–64. 10.1016/j.enzmictec.2018.09.003 30243388

[B30] Li H. Xi J. Donaghue A. G. Keum J. Zhao Y. An K. (2020). Synthesis and catalytic performance of polydopamine supported metal nanoparticles. Sci. Rep. 10 (1), 10416. 10.1038/s41598-020-67458-9 32591613 PMC7319955

[B31] Li Z. P. You S. Mao R. Xiang Y. Cai E. Deng H. (2022). Architecting polyelectrolyte hydrogels with Cu-assisted polydopamine nanoparticles for photothermal antibacterial therapy. Mater. Today Bio 15, 100264. 10.1016/j.mtbio.2022.100264 35517578 PMC9062430

[B32] Li T. Zhang J. Wen B. Wu Y. Che F. Guo Y. (2024). Dual-mode regulation of microbial cell membrane permeability for an enhanced microbial cuproptosis-like death pathway. Mater. Chem. Front. 9, 618–627. 10.1039/d4qm00935e

[B33] Li S. Li J. Xing J. Li L. Wang L. Wang C. (2025). Development and characterization of hyaluronic acid graft-modified polydopamine nanoparticles for antibacterial studies. Polymers 17 (2), 162. 10.3390/polym17020162 39861235 PMC11769165

[B34] Lin J. Li Y. Ge L. Zhang Y. Yuan S. Wang M. (2025). Photothermal enhanced ion release of ZIF-8/Ag/PDA for boosted antimicrobial performance. Inorg. Chem. Commun. 174, 114001. 10.1016/j.inoche.2025.114001

[B35] Liu Y. Liu R. Nie W. Yu L. Lyu S. Han Q. (2025). Enhanced corrosion resistance and photothermal antibacterial properties of MAO/PCL-PDA/NG composite coating on Mg alloy. Mater. Today Commun. 42, 111252. 10.1016/j.mtcomm.2024.111252

[B36] Lu Z. Xiao J. Wang Y. Meng M. (2015). *In situ* synthesis of silver nanoparticles uniformly distributed on polydopamine-coated silk fibers for antibacterial application. J. Colloid Interface Sci. 452, 8–14. 10.1016/j.jcis.2015.04.015 25909867

[B37] Lv Q. Cai Y. Yang R. Zhang L. Han Y. Marfavi Z. (2025). Efficient penetration and *in situ* polymerization of dopamine in biofilms for the eradication. Chem. Eng. J. 503, 158562. 10.1016/j.cej.2024.158562

[B38] Magalhães F. F. Pereira A. F. Freire M. G. Tavares A. P. M. (2022). New liquid supports in the development of integrated platforms for the reuse of oxidative enzymes and polydopamine production. Front. Bioeng. Biotechnol. 10, 1037322. 10.3389/fbioe.2022.1037322 36518198 PMC9742376

[B39] Marchesi D’Alvise T. Sunder S. Hasler R. Moser J. Knoll W. Synatschke C. V. (2023). Preparation of ultrathin and degradable polymeric films by electropolymerization of 3-Amino-l-tyrosine. Macromol. Rapid Commun. 44 (16), 2200332. 10.1002/MARC.202200332 35689352

[B40] Meng Y. Liu P. Zhou W. Ding J. Liu J. (2018). Bioorthogonal DNA adsorption on polydopamine nanoparticles mediated by metal coordination for highly robust sensing in serum and living cells. ACS Nano 12 (9), 9070–9080. 10.1021/acsnano.8b03019 30130385

[B41] Michalska M. Gambacorta F. Divan R. Aranson I. S. Sokolov A. Noirot P. (2018). Tuning antimicrobial properties of biomimetic nanopatterned surfaces. Nanoscale 10 (14), 6639–6650. 10.1039/c8nr00439k 29582025

[B42] Murray C. J. Ikuta K. S. Sharara F. Swetschinski L. Robles Aguilar G. Gray A. (2022). Global burden of bacterial antimicrobial resistance in 2019: a systematic analysis. Lancet 399 (10325), 629–655. 10.1016/S0140-6736(21)02724-0 35065702 PMC8841637

[B43] Nothling M. D. Bailey C. G. Fillbrook L. L. Wang G. Gao Y. McCamey D. R. (2022). Polymer grafting to polydopamine free radicals for universal surface functionalization. J. Am. Chem. Soc. 144 (15), 6992–7000. 10.1021/jacs.2c02073 35404602

[B44] Patel M. Corbett A. L. Vardhan A. Jeon K. Andoy N. M. O. Sullan R. M. A. (2023). Laser-responsive sequential delivery of multiple antimicrobials using nanocomposite hydrogels. Biomaterials Sci. 11 (7), 2330–2335. 10.1039/d2bm01471h 36892433

[B45] Peng S. Liu Y. Zhao W. Liu X. Yu R. Yu Y. (2025). Construction of pH-responsive hydrogel coatings on titanium surfaces for antibacterial and osteogenic properties. Front. Chem. 13, 1546637. 10.3389/fchem.2025.1546637 40051679 PMC11883361

[B46] Polaczek K. Olejnik A. Gumieniak J. Kramek A. Karczewski J. Siuzdak K. (2024). Lewis acid catalysis of polydopamine electropolymerisation as a tool for shaping its morphology and electrochemical properties. J. Mater. Sci. 59 (20), 9126–9149. 10.1007/s10853-024-09722-1

[B47] Pulingam T. Parumasivam T. Gazzali A. M. Sulaiman A. M. Chee J. Y. Lakshmanan M. (2022). “Antimicrobial resistance: prevalence, economic burden, mechanisms of resistance and strategies to overcome,”, 170. Elsevier B.V. 10.1016/j.ejps.2021.106103 34936936

[B48] Qi C. Zhang Y. Tu J. (2022). Facile synthesis of ε-poly- -lysine-conjugated ZnO@PDA as photothermal antibacterial agents for synergistic bacteria killing and biofilm eradication. Biochem. Eng. J. 186, 108569. 10.1016/j.bej.2022.108569

[B49] Ren S. Xiao X. Lv J. Lv S. Wang X. Liu R. (2024). Advances in the study of polydopamine nanotechnology in central nervous system disorders. Fronti. Mater. 11. 10.3389/fmats.2024.1396397

[B50] Saikawa G. I. A. Guidone G. H. M. Noriler S. A. Reis G. F. de Oliveira A. G. Nakazato G. (2024). Green-synthesized silver nanoparticles in the prevention of multidrug-resistant *Proteus mirabilis* infection and incrustation of urinary catheters BioAgNPs against *P. mirabilis* infection. Curr. Microbiol. 81 (4), 100. 10.1007/s00284-024-03616-w 38372801

[B51] Salam M. A. Al-Amin M. Y. Salam M. T. Pawar J. S. Akhter N. Rabaan A. A. (2023). Antimicrobial resistance: a growing serious threat for global public health. Healthc. Switz. 11 (Issue 13), 1946. 10.3390/healthcare11131946 37444780 PMC10340576

[B52] Schanze K. S. Lee H. Messersmith P. B. (2018). Ten years of polydopamine: current status and future directions. ACS Appl. Mater. Interfaces 10 (9), 7521–7522. 10.1021/acsami.8b02929 29510631

[B53] Sharma D. Hussain C. M. (2020). “Smart nanomaterials in pharmaceutical analysis,”, 13. Elsevier B.V, 3319–3343. 10.1016/j.arabjc.2018.11.007

[B54] Shumbula N. P. Nkabinde S. S. Ndala Z. B. Mpelane S. Shumbula M. P. Mdluli P. S. (2022). Evaluating the antimicrobial activity and cytotoxicity of polydopamine capped silver and silver/polydopamine core-shell nanocomposites. Arabian J. Chem. 15 (6), 103798. 10.1016/j.arabjc.2022.103798

[B55] Siciliano G. Monteduro A. G. Turco A. Primiceri E. Rizzato S. Depalo N. (2022). Polydopamine-coated magnetic iron oxide nanoparticles: from design to applications. Nanomaterials 12 (7), 1145. 10.3390/nano12071145 PMC9000600 35407264

[B56] Siddiqui I. Owais M. Husain Q. (2024). Antimicrobial effects of peptides from fenugreek and ginger proteins using Fe3O4@PDA-MWCNT conjugated trypsin by improving enzyme stability and applications. Int. J. Biol. Macromol. 282, 137197. 10.1016/j.ijbiomac.2024.137197 39489254

[B57] Singh R. Sharma A. Saji J. Umapathi A. Kumar S. Daima H. K. (2022). Smart nanomaterials for cancer diagnosis and treatment. Nano Converg. 9 (1), 21. 10.1186/s40580-022-00313-x 35569081 PMC9108129

[B58] Smies A. Wales J. Hennenfent M. Lyons L. Dunn C. Robbins J. (2022). Non-antibiotic antimicrobial polydopamine surface coating to prevent stable biofilm formation on satellite telemetry tags used in cetacean conservation applications. Front. Mar. Sci. 9, 989025. 10.3389/fmars.2022.989025

[B59] Soto-Garcia L. F. Guerrero-Rodriguez I. D. Hoang L. Laboy-Segarra S. L. Phan N. T. K. Villafuerte E. (2023). Photocatalytic and photothermal antimicrobial mussel-inspired nanocomposites for biomedical applications. Int. J. Mol. Sci. 24 (17), 13272. 10.3390/ijms241713272 37686076 PMC10488035

[B60] Souza A. L. de Oliveira A. V. de A. Ribeiro L. D. Moraes A. R. F. e. Jesus M. Santos J. (2025). Experimental and theoretical analysis of dopamine polymerization on the surface of cellulose nanocrystals and its reinforcing properties in cellulose acetate films. Polymers 17 (3), 345. 10.3390/polym17030345 39940547 PMC11821026

[B61] Talapko J. Meštrović T. Juzbašić M. Tomas M. Erić S. Horvat Aleksijević L. (2022). Antimicrobial peptides—Mechanisms of Action, antimicrobial effects and clinical applications. Antibiotics 11 (10), 1417. 10.3390/antibiotics11101417 36290075 PMC9598582

[B62] Tang K. W. K. Millar B. C. Moore J. E. (2023). Antimicrobial resistance (AMR). Br. J. Biomed. Sci. 80, 11387. 10.3389/bjbs.2023.11387 37448857 PMC10336207

[B63] Teunissen L. W. Smulders M. M. J. Zuilhof H. (2023). 19 nm-Thick Grafted-To polymer brushes onto optimized Poly(Dopamine)-Coated surfaces. Adv. Mater. Interfaces 10 (18), 2202503. 10.1002/admi.202202503

[B64] Tran H. Q. Batul R. Bhave M. Yu A. (2019). Current advances in the utilization of polydopamine nanostructures in biomedical therapy. Biotechnol. J. 14 (12), 1900080. 10.1002/BIOT.201900080 31293058

[B65] Varol H. S. Herberger T. Kirsch M. Mikolei J. Veith L. Kannan-Sampathkumar V. (2023). Electropolymerization of polydopamine at electrode-supported insulating mesoporous films. Chem. Mater. 35 (21), 9192–9207. 10.1021/acs.chemmater.3c01890 38027541 PMC10653081

[B66] Wang Z. Zou Y. Li Y. Cheng Y. (2020a). Metal-containing polydopamine nanomaterials: catalysis, energy, and theranostics. Small 16 (Issue 18), 1907042. 10.1002/smll.201907042 32220006

[B67] Wang Z. Zou Y. Li Y. Cheng Y. (2020b). Metal-containing polydopamine nanomaterials: catalysis, energy, and theranostics. Small 16 (Issue 18), 1907042. 10.1002/smll.20190042 32220006

[B68] Wang H. Ma J. Meng S. Zou H. Wang H. Zhou M. (2024). Functionalized mesoporous polydopamine nanocarrier with near-infrared laser-trigged NO release and photothermal effects for the killing of pathogenic bacteria. ACS Appl. Nano Mater. 7 (9), 10429–10441. 10.1021/acsanm.4c00865

[B69] Wang X. Huang J. Zhao J. Yue T. Shenyang W. Xu Y. (2025). pH-responsive cationic guar gum-based multifunctional hydrogel with silver nanoenzymes: combined photothermal antibacterial therapy and antioxidant properties for MRSA infected wound healing. Int. J. Biol. Macromol. 292, 139201. 10.1016/j.ijbiomac.2024.139201 39733885

[B70] Wang H H. Xu X. Mei X. Zeng D. Ying B. Yu Z. (2024). 3D-printed porous PEI/TCP composite scaffolds loaded with graphdiyne oxide on the surface for bone defect repair and near-infrared light-responsive antibacterial. Mater. Des. 237. 10.1016/j.matdes.2023.112569

[B71] Wang J J. Fang X. Yu G. Luo T. Xu Y. Xu C. (2024). Magnetic hybrid nanospindle with an unconventional force-thermal coupling antibacterial effect. Colloids Surfaces A Physicochem. Eng. Aspects 683, 133060. 10.1016/j.colsurfa.2023.133060

[B72] Wang L L chao B. Li Z. Ren feng K. jiang L. (2014). Electropolymerization of dopamine for surface modification of complex-shaped cardiovascular stents. Biomaterials, 35(27), 7679–7689. 10.1016/j.biomaterials.2014.05.047 24929615

[B73] Wang L L. Song K. Jiang C. Liu S. Huang S. Yang H. (2024). “Metal-coordinated polydopamine structures for tumor imaging and therapy,” in Advanced healthcare materials (John Wiley and Sons Inc). 10.1002/adhm.202401451 39021319

[B74] Wang R R. Dong Y. Zhang J. Hao L. Zhou L. Sun L. (2024). A multifunctional nanoplatform with dual-targeted antibacterial and cascaded immunomodulatory strategy for the treatment of bacterial keratitis. Chem. Eng. J. 498, 155323. 10.1016/j.cej.2024.155323

[B75] Wang S S. Meng F. Cao Z. (2024). Improving surface antimicrobial performance by coating homogeneous PDA-Ag micro–nano particles. Coatings 14 (7), 887. 10.3390/coatings14070887

[B76] Wang Y Y. Dong Y. Quan Y. Wackerow S. Abdolvand A. Zolotovskaya S. A. (2024). Hybrid antibacterial surfaces: combining laser-induced periodic surface structures with polydopamine-chitosan-silver nanoparticle nanocomposite coating. Adv. Mater. Interfaces 12, 2400660. 10.1002/admi.202400660

[B77] Wu Y. Liu X. Zhang X. Zhang S. Niu P. Gao H. (2023). Photothermal theranostics with glutathione depletion and enhanced reactive oxygen species generation for efficient antibacterial treatment. RSC Adv. 13 (33), 22863–22874. 10.1039/d3ra03246a 37520103 PMC10375255

[B78] Xiong C. Ulbricht M. (2024). Thermally responsive porous membranes with both switching wettability and permeability prepared *via* grafting-to and grafting-from methods. Chem. Eng. Res. Des. 209, 2–11. 10.1016/j.cherd.2024.07.025

[B79] Xu N. Huang Q. Shi L. Wang J. Li X. Guo W. (2023). A bioinspired polydopamine-FeS nanocomposite with high antimicrobial efficiency *via* NIR-Mediated fenton reaction. Dalton Trans. 52 (6), 1687–1701. 10.1039/d2dt03765c 36649112

[B80] Xu Q. Zhao Y. Yuan P. Ma X. Wang S. Li L. (2025). Functionalized microsphere platform combining nutrient restriction and combination therapy to combat bacterial infections. ACS Appl. Mater. and Interfaces 17 (2), 2966–2976. 10.1021/acsami.4c16610 39744763

[B81] Yang Z. You J. Zhai S. Zhou J. Quni S. Liu M. (2025). pH-responsive molybdenum disulphide composite nanomaterials for skin wound healing using “ROS leveraging” synergistic immunomodulation. Mater. Today Bio 31, 101481. 10.1016/j.mtbio.2025.101481 39925719 PMC11804827

[B82] Yoshida M. Lahann J. (2008). Smart nanomaterials. ACS Nano 2 (6), 1101–1107. 10.1021/nn800332g 19206325

[B83] Yu X. Zhao J. Fan D. (2022). A dissolving microneedle patch for antibiotic/enzymolysis/photothermal triple therapy against bacteria and their biofilms. Chem. Eng. J. 437, 135475. 10.1016/j.cej.2022.135475

[B95] Yusuf A. Almotairy A. R. Z. Henidi H. Alshehri O. Y. Aldughaim M. S. (2023). Nanoparticles as drug delivery systems: a review of the implication of nanoparticles’ physicochemical properties on responses in biological systems. Polymers 15 (7), 1596. 10.3390/POLYM15071596 37050210 PMC10096782

[B84] Zeng Q. Qian Y. Huang Y. Ding F. Qi X. Shen J. (2021). Polydopamine nanoparticle-dotted food gum hydrogel with excellent antibacterial activity and rapid shape adaptability for accelerated bacteria-infected wound healing. Bioact. Mater. 6 (9), 2647–2657. 10.1016/j.bioactmat.2021.01.035 33665497 PMC7890098

[B85] Zhang H. Lu M. Jiang H. Wu Z. Y. Zhou D. D. Li D. Q. (2020). Tyrosinase-mediated dopamine polymerization modified magnetic alginate beads for dual-enzymes encapsulation: preparation, performance and application. Colloids Surfaces B Biointerfaces 188, 110800. 10.1016/j.colsurfb.2020.110800 31958620

[B86] Zhang H H. Zou Y. Lu K. Wu Y. Lin Y. Cheng J. (2024). A nanoplatform with oxygen self-supplying and heat-sensitizing capabilities enhances the efficacy of photodynamic therapy in eradicating multidrug-resistant biofilms. J. Mater. Sci. Technol. 169, 209–219. 10.1016/j.jmst.2023.07.001

[B87] Zhang R R. Zhang W. Zhu Q. Nie Q. Zhang S. Zhang Y. (2024). Engineering polydopamine-functionalized NH2-MIL-125 (ti) for tetracycline degradation and antibacterial applications. Surfaces Interfaces 54, 105188. 10.1016/j.surfin.2024.105188

[B88] Zhang X X. Liu Y. Zhang X. Tang M. Xi W. Wei J. (2024). Antimicrobial GL13K peptide-decorated ZnO nanoparticles to treat bacterial infections. Langmuir 40, 25042–25050. 10.1021/acs.langmuir.4c03206 39540901

[B89] Zhao B B. Shi X. Qiu H. Chen K. (2024). Design and application of polyurethane-polydopamine/Ag double-shell microcapsules for enhanced photothermal conversion and incremental energy storage. Sustain. Mater. Technol. 40, e00895. 10.1016/j.susmat.2024.e00895

[B90] Zhao X. Xu Z. Wei Z. Sun Y. Zhou Q. (2023). Nature-inspired mechano-bactericidal nanostructured surfaces with photothermally enhanced antibacterial performances. Prog. Org. Coatings 182, 107599. 10.1016/j.porgcoat.2023.107599

[B91] Zhao Q Q. Zhou Y. Zhang Q. Qu X. Jiang Y. Wu S. (2024). Cobalt doped Prussian blue modified hollow polydopamine for enhanced antibacterial therapy. Nanotechnology 35 (36), 365101. 10.1088/1361-6528/ad53d2 38834038

[B92] Zhao Y Y. Zhan K. Geng P. Jiang S. (2024). Polydopamine-assisted decoration of silver nanoparticles on gold nanorods for photothermal and chemical antimicrobial applications. New J. Chem. 49, 624–631. 10.1039/d4nj04434g

[B93] Zhou Y. Zhang Q. Xia Z. Wang Z. Liu L. Jiang Y. (2024). Mixed-charge cellulose nanocrystal modified cellulose acetate membrane with endotoxin scavenging ability and antibacterial properties. J. Appl. Polym. Sci. 141 (33), e55834. 10.1002/app.55834

