# Supplementary figures and images for: Recent progress of polydopamine nanoparticles as advanced antimicrobial nanomaterials
Source: Front Bioeng Biotechnol. 2025 Oct 17;13:1678136. doi: 10.3389/fbioe.2025.1678136 (PMC12575315; doi:10.3389/fbioe.2025.1678136)

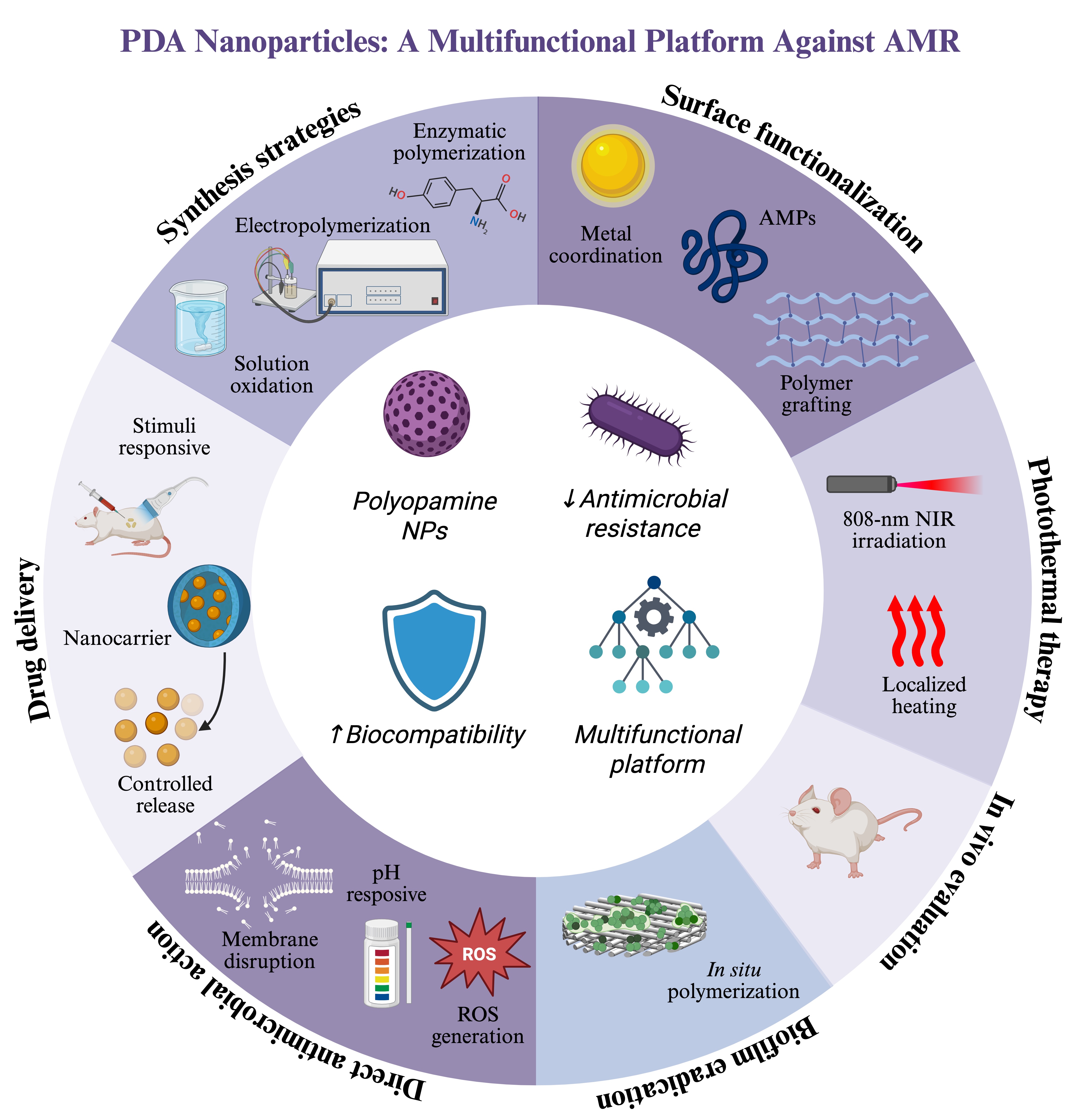

Supplement: Supplementary file 1 [file Image1.jpeg]
